# Conserved function of a RasGEF‐mediated pathway in the metabolic compensation of the circadian clock

**DOI:** 10.1111/febs.70122

**Published:** 2025-05-02

**Authors:** Orsolya Sárkány, Anita Szőke, Aladár Pettkó‐Szandtner, Eszter Éva Kálmán, Michael Brunner, Norbert Gyöngyösi, Krisztina Káldi

**Affiliations:** ^1^ Department of Physiology Semmelweis University Budapest Hungary; ^2^ Biological Research Centre Szeged Hungary; ^3^ Department of Molecular Biology Semmelweis University Budapest Hungary; ^4^ Biochemistry Center Heidelberg University Heidelberg Germany

**Keywords:** BMAL1, cAMP, circadian rhythm, ERK, metabolic compensation, *Neurospora crassa*, RAS pathway, RasGEF, SOS1

## Abstract

Metabolic compensation of the circadian clock ensures endogenous timing across a broad range of nutrient conditions, enabling organisms to adapt efficiently to recurrent environmental changes, even during nutrient scarcity. In this study, we have identified a novel clock‐controlled gene, *rasgef* (Rat Sarcoma Guanine Nucleotide Exchange Factor), that plays a crucial role in modulating the circadian clock under starvation conditions in the circadian model organism *Neurospora crassa*. The gene product, RasGEF—a nucleotide exchange factor for the small G protein RAS2P (Rat Sarcoma 2 Protein)—displays glucose‐dependent phosphorylation and localization. We show that deletion of *rasgef* hinders metabolic compensation of the circadian clock to glucose‐depleted conditions and disrupts the rhythmic expression of the output gene *ccg2*. Furthermore, we demonstrate in osteosarcoma cells that the period of the mammalian clock is also compensated across a wide range of extracellular glucose levels and adaptation of the clock to glucose‐starved conditions depends on the RasGEF homolog SOS1 (Son of Sevenless 1) and its downstream signaling component ERK (Extracellular Signal‐Regulated Kinase). Our results suggest a conserved role of RasGEF‐mediated signaling in the maintenance of circadian rhythm under glucose‐limited conditions.

## Graphical abstract

Metabolic compensation of the circadian clock is essential for stabilizing the period of endogenous timekeeping in environments with significant nutrient fluctuations. In this study, we demonstrate that RasGEF (SOS1)‐mediated signaling, which is influenced by metabolic conditions, supports the robust function of the circadian clock under low glucose conditions in both the circadian model organism *Neurospora crassa* and mammalian cells.

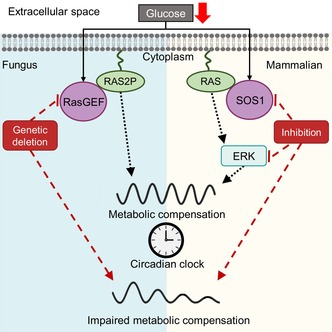

**Abbreviations**
ANOVA: analysis of variance
*bd*
: *band*
BMAL1: brain and muscle ARNT‐like 1
*ccg*
: *clock‐controlled gene*
*ccg‐2*
: *clock‐controlled gene 2*
CDC25: cell division cycle 25 phosphatase
CIP: calf intestinal phosphatase
CK: casein kinase
CLOCK: circadian locomotor output cycles kaput
CRY: cryptochrome
CT: circadian time
D/L: dark/light
DD: constant darkness
DMEM: Dulbecco's modified Eagle medium
DMSO: dimethyl sulfoxide
ERK: extracellular signal‐regulated kinase
FBS: fetal bovine serum
FC: fold change
FDR: false discovery rate
Fisher's LSD: Fisher's least significant difference
FRH: FRQ‐interacting RNA helicase
FRQ: FREQUENCY
*gna‐3*
: *G protein alpha subunit‐3*
HCD: higher‐energy collisional dissociation
*his3*
: *histidine biosynthesis gene 3*
IP: immunoprecipitation
KO: knockout
L/D: light/dark
LC: loading control
LC–MS/MS: liquid chromatography–tandem mass spectrometry
LL: constant light
MAPK: mitogen‐activated protein kinase
MEF: mouse embryonic fibroblast
MEK1/2: MAPK/ERK kinase 1/2
MSMS: tandem mass spectrometry
ns: not significant
PER: PERIOD
PKA: protein kinase A
PP2A: protein phosphatase 2A
RAS GTPases: RAS guanosine triphosphatases
*ras*
: *rat sarcoma oncogene*
RAS2P: Rat sarcoma 2 protein
RasGEF: RAS guanine nucleotide exchange factor
RGB‐1: regulatory subunit of protein phosphatase 2A
ROS: reactive oxygen species
SDS: sodium dodecyl sulfate
SOS1 and 2: Son of Sevenless 1 and 2
TBS: tris‐buffered saline
TTFL: transcriptional‐translational feedback loop
Tukey's HSD: Tukey's honest significant difference
U2OS: Uppsala 2 osteosarcoma
WC‐1: white collar 1
WC‐2: white collar 2
WCC: white collar complex
ZT: Zeitgeber time

## Introduction

Circadian clocks orchestrate the timely adjustment of physiological and biochemical processes to the daily environmental fluctuations. Numerous studies have demonstrated that a robust circadian rhythm offers survival advantages across various evolutionary levels [1, 2, 3, 4, 5]. In humans, disruptions of circadian rhythms are frequently associated with an increased risk of cardiovascular and metabolic diseases, as well as certain cancers [6, 7].

The core of eukaryotic circadian clocks is a cell‐autonomous transcriptional‐translational feedback loop (TTFL). This TTFL involves circadian transcription factors that regulate the expression of their own inhibitors, creating a self‐sustaining rhythm of clock component expression and downstream circadian output pathways [8, 9, 10, 11, 12, 13, 14]. Although the specific circadian transcription factors and their inhibitors differ between *Neurospora* and mammals, the TTFLs are similarly organized and regulated by casein kinase 1 (CK1).

In *Neurospora*, the white collar complex (WCC) formed by the transcription factors WC‐1 and WC‐2 activates the rhythmic expression of the *Frequency* (*frq*) gene. The FRQ protein interacts with FRH (FRQ‐interacting RNA helicase) and CK1, leading to the inhibition of the WCC through phosphorylation [15, 16, 17]. Over the course of a circadian cycle, FRQ also undergoes multiple phosphorylations and subsequently becomes degraded [18, 19, 20]. In addition to CK1a, other kinases and phosphatases also regulate the activity of both the WCC and FRQ [21]. The WCC, as the major photoreceptor of *Neurospora*, transduces light information to the oscillator and entrains the clock accordingly [22, 23].

In mammals, the heterodimeric transcription factor BMAL1/CLOCK drives the rhythmic expression of the *Period* (*Per*) and *Cryptochrome* (*Cry*) genes. PER proteins form complexes with CRY and CK1, which inactivate BMAL1/CLOCK. Throughout the circadian cycle, PER proteins are gradually phosphorylated by CK1 and eventually degraded [24].

Circadian clocks are closely linked to metabolism, rhythmically regulating metabolic pathways while also being influenced by nutrients and metabolic signals. In humans, circadian rhythm dysfunctions—such as those caused by shift work, jet lag, or various sleep disorders—are associated with an elevated risk of metabolic disturbances, like obesity, metabolic syndrome, and type 2 diabetes. A central question in circadian research is understanding how the speed of the TTFL, and thus, the period of the circadian cycle is maintained across varying nutrient levels. This mechanism—called nutritional or metabolic compensation—has recently been shown for the mammalian clock as well; however, only at normal average and supraphysiological extracellular glucose levels [25]. Metabolic compensation has been studied in most detail in *Neurospora* [26, 27, 28, 29, 30], but even in this case, the molecular mechanism is only partially understood. In a very recent study, we showed that the amount of the WCC and phosphorylation of FRQ undergo characteristic modifications upon glucose withdrawal, but the level and rhythm of *frq* RNA are kept stable and clock‐controlled output remains robust [31]. Our results suggested that the cAMP/PKA‐mediated pathway is central in adapting clock function to changes in glucose supply. The RAS‐mediated pathway plays a central role in glucose sensing in fungi. We recently proposed that in *Neurospora*, RAS2 acting via a cAMP‐dependent pathway links the circadian clock to glucose‐dependent signaling and thereby is an important component of the nutritional compensation of the period [28].

The circadian rhythm of *Neurospora* is modulated by RAS1, as well [32]. When *ras‐1* ^*bd*^ strains, which carry the activating mutation of *ras‐1*, are grown on race tubes, rhythmic conidiation is detected as a spatially restricted banding pattern, whereas *ras* ^*wt*^ strains show rhythmic spore formation only on specific media, such as those containing the ROS generator menadione [32, 33].

In this study, we examined the involvement of a RAS guanine nucleotide exchange factor (RasGEF) in the regulation of the circadian clock. Our findings in *Neurospora* indicate that both expression and phosphorylation of this RasGEF are dependent on glucose. We identify RAS2P, a signaling factor distinct from RAS2, as the primary monomeric G protein partner of RasGEF. Our data reveal a bidirectional interaction between RasGEF and the circadian clock; the clock controls *rasgef* expression, and RasGEF affects metabolic compensation of the circadian phase and period under low glucose conditions. We show that the circadian period of human U2OS cells is also compensated across a broad range of extracellular glucose levels, including hypoglycemic conditions. Furthermore, our results suggest a role for RasGEF(SOS1)‐mediated signaling in maintaining clock function in nutrient‐deprived mammalian cells as well.

## Results

### A RasGEF is involved in the regulation of the conidiation rhythm in *Neurospora crassa*

**Fig. 1:**
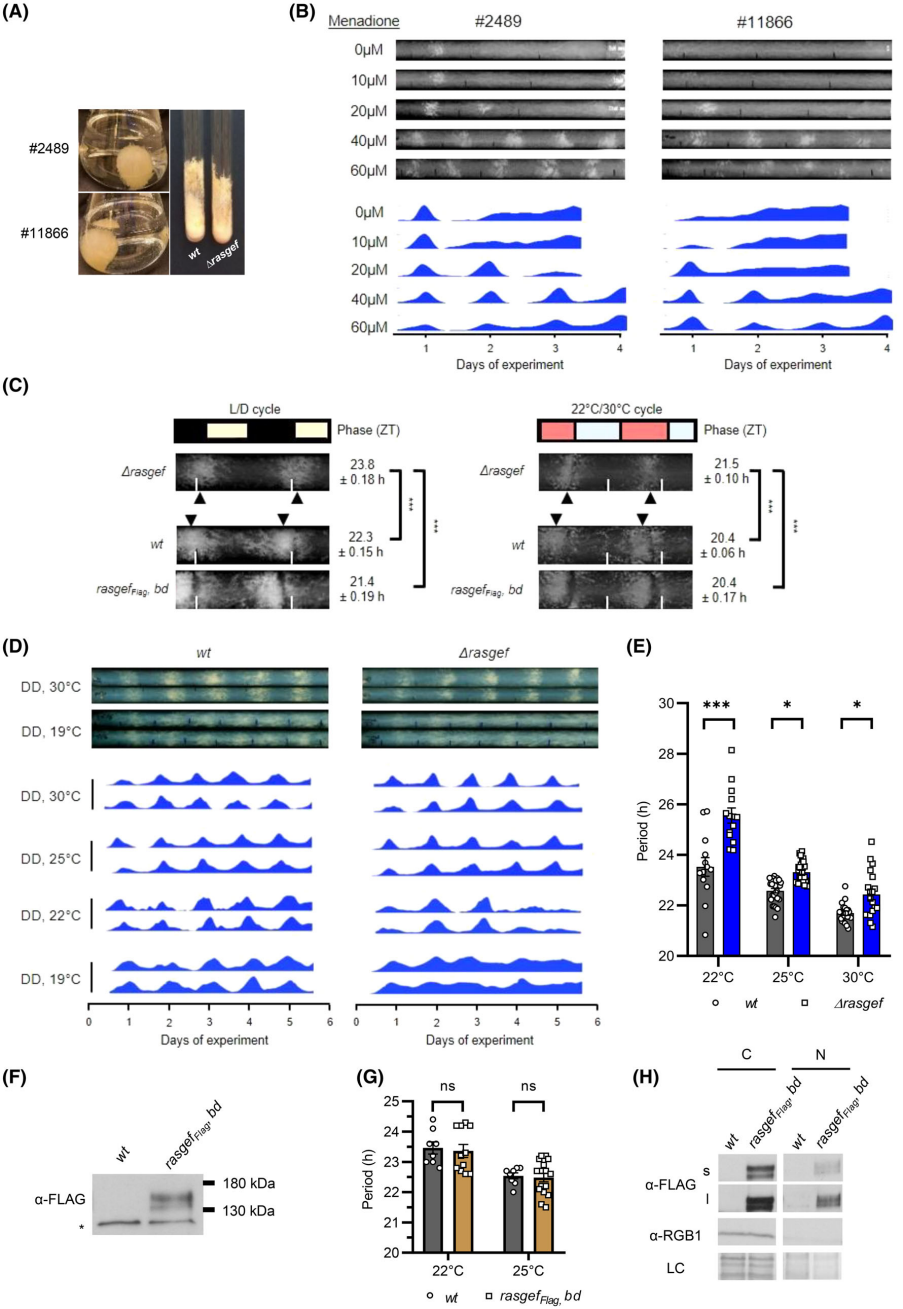
RasGEF controls the conidiation rhythm in *Neurospora crassa*. (A) The growth phenotype of *Δrasgef* is similar to that of the *wt* strain both in liquid (left panel) and on solid (right panel) medium. Representative cultures are shown (*n* = 30). (B) Banding is less sensitive to menadione in the *rasgef* deletion strain (FGSC #11866) than in *wt* (FGSC #2489). Race tubes with the indicated concentrations of menadione were inoculated with the indicated strains. Following an incubation in constant light (LL) for 2 days, race tubes were transferred to constant darkness (DD). Upper panels: Representative race tubes are shown (*n* = 4). Lower panels: Averaged densitometric curves of race tube duplicates from a representative experiment are shown. As menadione affects growth rate, different numbers of days could be displayed on the densitometric plots. (C) The phase of banding is delayed in *Δrasgef* compared to *wt* under both light/dark (L/D) (left panel) and temperature cycles (right panel) and expression of RasGEF_Flag_ rescues the phenotype. Race tubes were incubated under 12/12 h L/D or 22/30 °C temperature cycles, as indicated. The white lines refer to the same time points within the panel. For better comparison of the conidial bands' positions, images were slightly adjusted to ensure similar daily growth distances in the parallel samples. Conidiation peaks are indicated by arrows and given in Zeitgeber time (ZT) (*n* (L/D) = 23 (*rasgef*_*Flag*_, *bd*), 66 (*Δrasgef*) and 59 (*wt*); *n* (22/30 °C) = 8). (D) The conidiation rhythm of *Δrasgef* shows dampening at low temperatures. Race tubes were incubated in DD and at the indicated temperatures. Upper panels: Images of representative tubes are shown. Lower panels: Densitometric analysis of the race tubes incubated at the indicated temperatures. (E) Impaired temperature compensation of the period in *Δrasgef*. Race tubes from the experiment described in (D) were analyzed with the ChronOSX program (*n* (22 °C) = 13 (*wt*) and 14 (*Δrasgef*); *n* (25 °C) = 26 (*wt*) and 26 (*Δrasgef*); *n* (30 °C) = 21 (*wt*) and 20 (*Δrasgef*). (F) Expression of RasGEF_Flag_ in *Δrasgef*. The indicated strains were grown in liquid cultures in LL, and whole cell extracts were analyzed by western blotting using an anti‐FLAG antibody (*n* = 6). Asterisk, unspecific band. (G) Expression of RasGEF_Flag_ rescues the period in *Δrasgef*. Experiments were performed with the indicated strains as described in (D) (*n* (22 °C) = 8 (*wt*) and 11 (*Δrasgef*); *n* (30 °C) = 8 (*wt*) and 16 (*Δrasgef*). (H) RasGEF is localized to both the nucleus and the cytosol. Subcellular fractionation with the indicated strains was performed. RasGEF_Flag_ in the cytosolic (C) and the nuclear (N) fractions was detected. A representative western blot is shown (*n* = 5); RGB‐1 was detected as a cytosolic marker. s, short; l, long exposure; LC, loading control. All data were presented as mean ± SEM. Statistical analysis was performed using one‐way ANOVA followed by Tukey's HSD test (C) and two‐way ANOVA followed by Fisher's LSD test (E, G). ns, not significant; *P < 0.05; ***P < 0.001.

Building on data indicating an interplay between RAS‐mediated signaling and the circadian rhythm in *Neurospora* [28, 32], we aimed to investigate the role of RasGEF activity in the regulation of the circadian clock and its metabolic compensation. *Neurospora* contains four RasGEF domain‐encoding genes, two of which (NCU 06500 and NCU 09758) include all conserved domains found in CDC25 [34], a RasGEF central in glucose sensing in *Saccharomyces cerevisiae* [35]. A knockout strain homozygous for NCU 09758 deletion (FGSC #11866) could be generated during the *Neurospora* Genome project [36], while a deletion strain for NCU 6500 is available only as a heterokaryon. Hence, we focused our investigation on the RasGEF encoded by NCU 09758. The overall morphology of the NCU 09758 knockout strain (FGSC #11866) was similar to that of the corresponding *rasgef* ^+^ control strain (FGSC #2489) both on solid medium and in liquid cultures (Fig. 1A). However, when grown on race tube medium, the *rasgef* knockout strain exhibited a slightly faster growth compared to the control across different temperatures (Fig. S1A). To visualize rhythmic banding, menadione‐containing medium was used. Sensitivity to menadione of the mutant was, however, lower compared to the control, suggesting that the absence of RasGEF affects the clock output (Fig. 1B). When the strains were entrained to light/dark (L/D) or temperature cycles, the phase of the conidiation in FGSC #11866 was delayed (Fig. S1B), further indicating an impact of RasGEF on circadian regulation.

To better visualize the conidiation rhythm, we crossed FGSC #11866 with the *ras‐1* ^*bd*^ strain. For convenient reading, in the following sections of the manuscript this strain and the control *bd* strain will be referred to as *Δrasgef* and *wt*, respectively. Like FGSC #11866, *Δrasgef* also exhibited a delayed conidiation phase under both L/D and temperature cycles compared to *wt* (Fig. 1C), suggesting that enhanced activity of RAS‐1 cannot compensate for the lack of RasGEF function. The robust conidiation in the *bd* background also allowed the examination of the rhythm in DD and at lower temperatures. Compared to *wt*, banding in *Δrasgef* was more sensitive to low temperatures, leading to a strongly dampened rhythm at 19 °C (Fig. 1D). In the temperature range between 22 and 30 °C, *Δrasgef* displayed a longer period than *wt*, with a more pronounced difference at lower temperatures, suggesting an impaired temperature compensation in the absence of RasGEF (Fig. 1E).

To generate a rescue strain and detect RasGEF at the protein level, we expressed a Flag‐tagged version of RasGEF in the *Δrasgef* strain under the control of the *ccg1* promoter (*rasgef*_Flag_, *bd*). Western analysis using an anti‐FLAG antibody detected multiple bands in the size range corresponding to the calculated molecular weight (136 kDa) (Fig. 1F), suggesting that RasGEF_Flag_ was post‐transcriptionally modified. Expression of RasGEF_Flag_ restored the wild‐type phase in both light–dark and temperature cycles, as well as the wild‐type period in constant darkness (DD), confirming that the deletion of *NCU 09758* was responsible for the circadian phenotype of *∆rasgef* (Fig. 1C,G). Subcellular fractionation revealed that RasGEF_Flag_ accumulated in both the cytosol and the nucleus of *Neurospora* (Fig. 1H), similar to CDC25 in budding yeast [37, 38, 39].

### The *Neurospora* clock function is modified by RasGEF in a glucose‐dependent manner

**Fig. 2:**
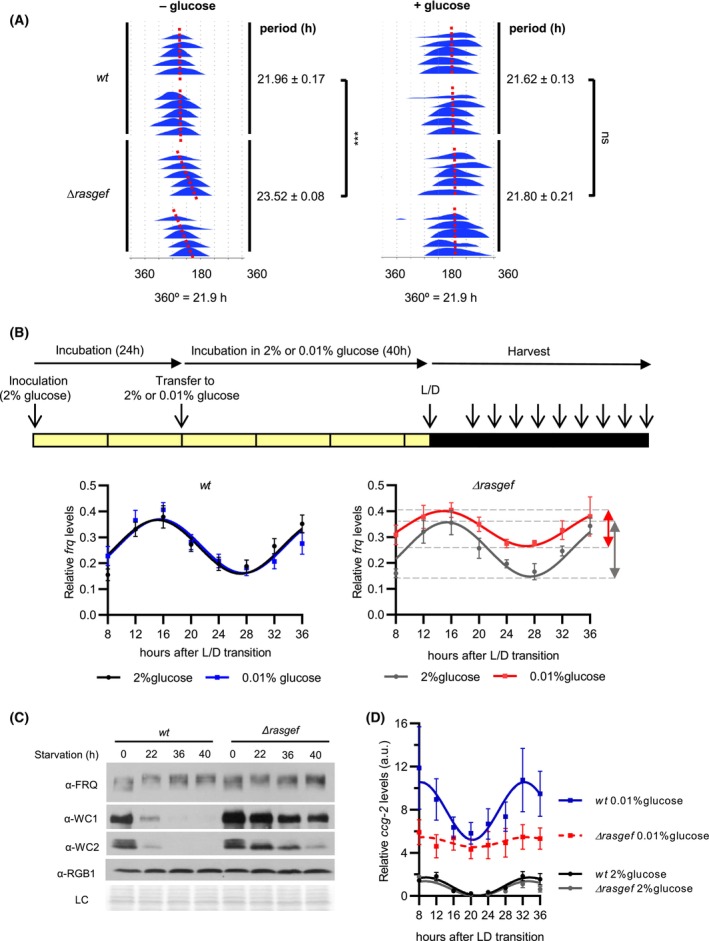
Starvation‐dependent clock phenotype of *Δrasgef*. (A) Period of the conidiation rhythm depends on glucose in *Δrasgef*. Race tubes containing no (− glucose) or 0.3% glucose (+ glucose) were inoculated with the indicated strains and, following 24 h of incubation in constant light, transferred to constant darkness (25 °C). Conidial densities of consecutive cycles are plotted below each other. Plots show representative individual race tubes (*n* = 4). (B) Amplitude of *frq* oscillation is reduced and *frq* levels are increased in starved *Δrasgef* cells as compared to standard growth conditions. The upper panel shows the schematic outline of the experimental design. Following an incubation of the indicated strains in standard liquid medium for 24 h, mycelia were transferred to standard (black and gray curves; lower panels) or starvation medium (blue and red curves; lower panels) in constant light (LL). After 40 h of incubation in LL, cultures were transferred to constant darkness (DD). Samples were harvested at the indicated time points and RNA levels were quantified. Sine curves were fitted to the time series (*wt*, 2% glucose, *n* = 8 (8, 16, 32 h), 9 (12, 28 h) and 10 (20, 24, 36 h); *wt*, 0.01% glucose, *n* = 9 (8, 12 h), 7 (16, 28, 32 h), 10 (20 h) and 8 (24, 36 h); *Δrasgef*, 2% glucose, *n* = 7 (8, 16, 24, 36 h) and 6 (12, 20, 28, 32 h); *Δrasgef*, 0.01% glucose, *n* = 7 (8, 24, 28 h) and 6 (12, 16, 20, 32, 36 h)). (C) Response of WCC expression to starvation is different in *Δrasgef* and *wt*. Mycelial discs were incubated in LL for 24 h in standard liquid medium and then transferred to starvation medium (time point 0). Samples were harvested at the indicated time points. Cell extracts were analyzed by western blotting (*n* = 2). RGB‐1 and Ponceau staining are shown as loading controls (LC). (D) *ccg‐2* does not display rhythmic expression in starved *Δrasgef*. *ccg‐2* RNA levels in samples prepared as described in (B) were quantified and analyzed (*wt*, 2% glucose, *n* = 7 (8, 12, 16, 36 h), 6 (20, 24, 32 h) and 5 (28 h); *wt*, 0.01% glucose, *n* = 5 (8, 28 h), 6 (12, 20, 24 h), 8 (16, 32 h) and 7 (36 h); *Δrasgef*, 2% glucose, *n* = 7 (8 h), 8 (12, 16, 20 h), 6 (24 h) and 5 (28, 32, 36 h); *Δrasgef*, 0.01% glucose, *n* = 6)). All data were presented as mean ± SEM. Statistical analysis was performed using two‐way ANOVA followed by Tukey's HSD test (A) and cosinor analysis (B, D). Solid line indicates significant fitting, whereas dashed line indicates no significant fitting to a 24‐h sinusoidal function. ns, not significant; ***P < 0.001.

As RAS‐mediated signaling affects metabolic compensation of the circadian rhythm in *Neurospora crassa* [28], we examined whether the impact of *rasgef* deletion on the conidiation rhythm is dependent on glucose. In glucose‐containing medium, the phase of conidiation was delayed in both *Δrasgef* and *wt* compared to no‐glucose conditions. However, the period difference between *Δrasgef* and *wt* observed in minimal medium was absent in the glucose‐containing medium (Fig. 2A). The glucose‐dependent circadian phenotype of *Δrasgef* suggests that the lack of RasGEF function affects the ability of the circadian clock to adapt to starvation.

We then measured the circadian expression profile of the clock gene *frq*. In *wt*, the oscillation of *frq* RNA levels was identical under starvation and standard glucose conditions (Fig. 2B, left panel), indicating that the core clock was metabolically compensated. Under standard conditions, expression levels and oscillation of *frq* RNA in *Δrasgef* was similar to that in the *wt*. However, in starved *Δrasgef* cultures *frq* RNA was expressed at a higher level and oscillated with a lower relative amplitude (peak : trough ratio), suggesting a compromised metabolic compensation of the circadian clock in the absence of RasGEF (Fig. 2B, right panel).

When light‐grown *wt* cultures were shifted from standard to starvation medium, FRQ was hyperphosphorylated, as reported previously [31] and WCC levels decreased substantially in response to glucose withdrawal (Fig. 2C, left part). FRQ was also hyperphosphorylated in response to starvation in *Δrasgef*. However, WC‐1 and WC‐2 were slightly overexpressed in *Δrasgef* and their levels remained higher than in *wt* upon glucose starvation.

Next, we investigated the circadian expression of the glucose‐repressible gene *ccg‐2* which belongs to the best characterized output genes of the *Neurospora* clock [40, 41, 42, 43]. *ccg‐2* encodes the conidial protein hydrophobin that is crucial for effective spore dispersal. In standard medium, *ccg‐2* RNA displayed expression with similar amplitude and phase in *wt* and *Δrasgef* (Fig. 2D). Under starvation conditions, *ccg‐2* was expressed at a higher level in both strains; however, the increase was less pronounced in the mutant than in *wt*. Furthermore, while *ccg‐2* exhibited rhythmic expression during starvation in the *wt*, the rhythm was severely blunted in *Δrasgef* (Fig. 2D). Overall, our observations indicate that in the absence of RasGEF, adaptation of the circadian regulation to glucose depletion is impaired.

### *rasgef* expression is rhythmic and affected by both light and glucose availability

**Fig. 3:**
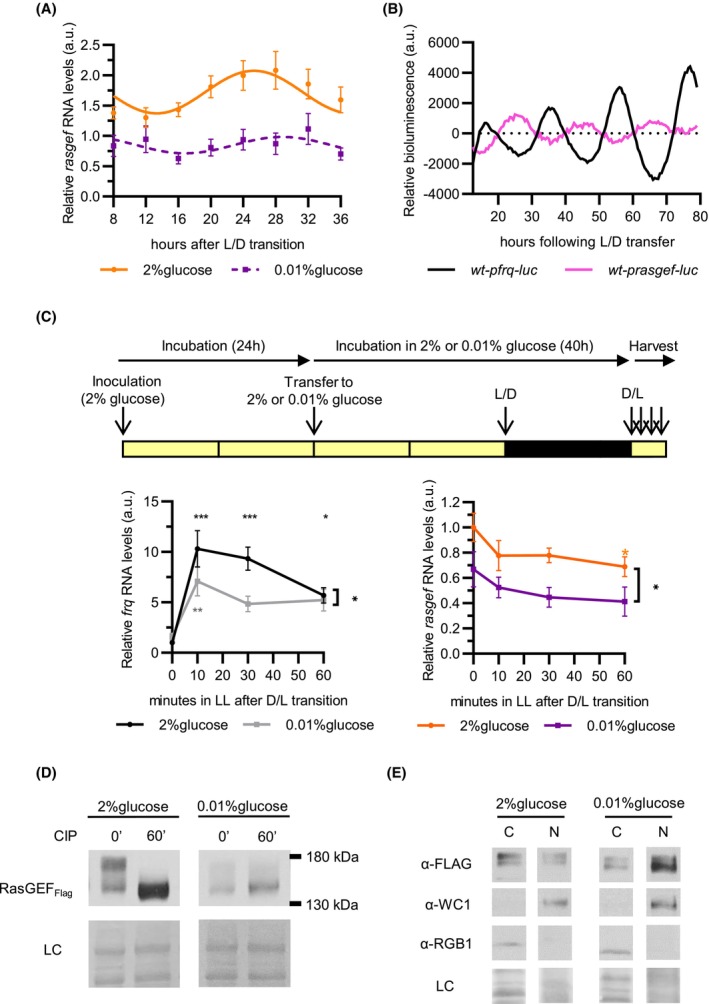
Analysis of *rasgef* expression. (A) *rasgef* expression is rhythmic under standard conditions. *rasgef* RNA levels were quantified in the *wt* samples from the experiment described in Fig. 2B (*n* = 7, except at 2% glucose, 16 and 36 h: *n* = 6). (B) Activity of the *rasgef* promoter shows circadian oscillation in antiphase to *frq* expression. *In vivo* luciferase assay was performed with the indicated strains. Time‐dependent changes in luciferase activity are shown by averaged curves (*n* = 6). (C) *rasgef* expression is repressed by light. Upper panel: Mycelial discs of the *wt* strain were incubated in standard liquid medium for 24 h, then transferred to either standard or starvation medium. Following an incubation in constant light (LL) for 24 h, cultures were transferred to constant darkness (DD) for 16 h and then to constant light (dark/light transition – D/L transition). Samples were harvested at the indicated time points after light on. Relative *frq* (left panel) and *rasgef* (right panel) RNA levels were normalized to that measured at time point 0 under standard conditions (*n* = 8). (D) RasGEF is a phosphoprotein. *rasgef*_Flag_, *bd* cultures were grown in standard liquid medium for 24 h in LL and then transferred to either standard or starvation medium. After 40 h incubation in LL, cultures were harvested and protein lysates were prepared, and analyzed by western blotting using an anti‐FLAG antibody. When indicated, the protein lysates were treated with λ phosphatase (CIP) for 60 min (*n* = 4). Ponceau staining is shown as a loading control (LC). (E) Both phosphorylation and subcellular localization of RasGEF_Flag_ are dependent on glucose. Experiments were performed with the *rasgef*_Flag_, *bd* strain as described in Fig. 1H. Nuclear (N) and cytosolic (C) fractions were analyzed by western blotting. RGB‐1 is shown as a cytosol marker, WC‐1 as a protein dominantly localized to the nucleus, and Ponceau staining was used as a loading control (LC) (*n* = 6). The data were presented as mean (B) or mean ± SEM (A, C). Statistical analysis was performed using cosinor analysis (A) and repeated measurement ANOVA followed by Fisher's LSD test (C), which revealed a significant group effect (C). The solid line indicates significant fitting, whereas the dashed line indicates no significant fitting to a 24‐h sinusoidal function. ns, not significant; *P < 0.05; **P < 0.005; ***P < 0.001.

We followed *rasgef* expression in DD throughout the circadian cycle (Fig. 3A). In the standard medium, the average *rasgef* RNA levels were about two times higher compared to the starved cultures and displayed significant oscillation, whereas only a nonsignificant fluctuation was detected under starvation conditions. To further examine the control of *rasgef* transcription by the circadian clock, we generated a strain (*wt*‐*prasgef‐luc*) expressing luciferase under the control of the *rasgef* promoter. *wt‐prasgef‐luc* exhibited rhythmic luciferase activity which was in antiphase compared with the luciferase signal detected in *wt‐pfrq‐luc* (Fig. 3B). These data indicate that *rasgef* is controlled by an evening‐specific promoter.

Next, light response of *rasgef* expression was examined. Dark‐grown cultures were transferred to light, and *rasgef* and *frq* transcript levels were determined and compared. While *frq* expression was strongly induced by light and its kinetics depended on glucose (Fig. 3C, left panel), as reported previously [31], *rasgef* levels slightly decreased under both culture conditions (Fig. 3C, right panel). These data indicate that *rasgef* is a light‐repressible gene.

CDC25, the yeast homolog of RasGEF, is regulated by phosphorylation [44, 45, 46]. Since RasGEF_Flag_ was detected as two dominant bands on the western blot, we examined phosphorylation of RasGEF_Flag_. When protein extracts from *rasgef*_Flag_ were treated with alkaline phosphatase, a protein form with higher electrophoretic mobility was detected, suggesting that RasGEF is a phosphoprotein (Fig. 3D). In standard medium, the hyperphosphorylated RasGEF species dominated, whereas the hypophosphorylated form was more abundant under starvation conditions. Subcellular fractionation revealed that under high glucose conditions, where hyperphosphorylation is prevalent, RasGEF_Flag_ is primarily cytoplasmic. Conversely, in low glucose conditions, RasGEF_Flag_ becomes hypophosphorylated and accumulates in the nucleus (Fig. 3E). These findings suggest that glucose availability modulates both the posttranslational modification and subcellular localization of RasGEF in *Neurospora*, similar to the regulation of CDC25 in yeast [44, 45, 46].

### RasGEF activity is linked to cAMP signaling and interacts with the monomeric G protein RAS2P

**Fig. 4:**
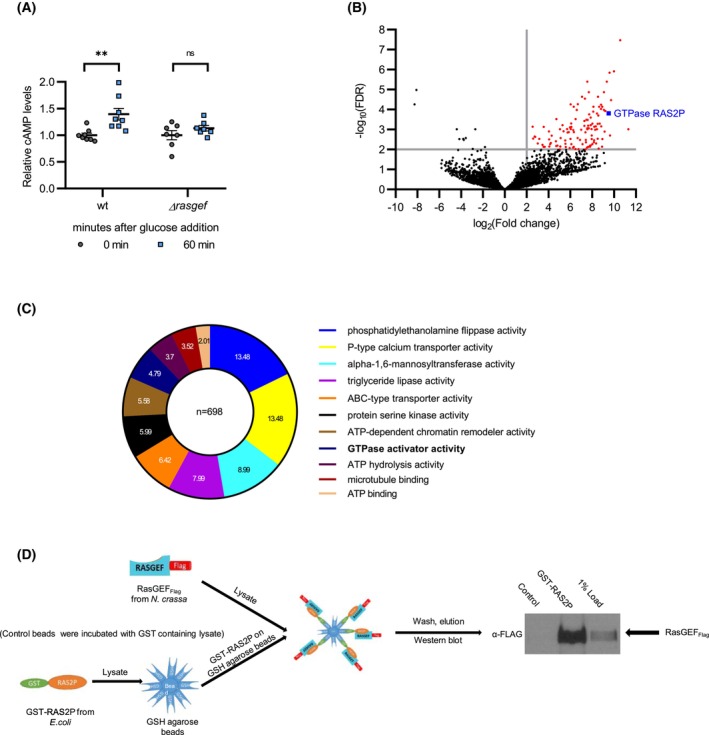
RasGEF affects cAMP signaling and interacts with RAS2P. (A) Glucose‐induced cAMP elevation is impaired in *Δrasgef*. Indicated strains were grown as described in Fig. 3D. Following the 40‐h incubation in the starvation medium, 2% glucose was added to the cultures. Samples were harvested at the indicated time points, and cAMP levels were determined in the whole cell lysates and normalized to that measured at time point 0 (*n* = 7 (*Δrasgef*) and 8 (*wt*)). (B) Volcano plot visualization of the data of the mass spectrometry analysis for the identification of possible interaction partners of RasGEF. Protein lysate of *rasgef*_Flag_ was subjected to affinity purification using an anti‐FLAG antibody. *wt* (#2489) lysate served as control. Following mass spectrometry analysis, RasGEF_Flag_‐associated proteins were identified. Red points on the plot indicate proteins with log_2_(FDR) ≥ 2 and log_10_FC ≥ 2. The blue point shows the position of RAS2P (*n* = 8 (*rasgef*_*Flag*_), *n* = 5 (#2489)). (C) Functional enrichment analysis of the mass spectrometry data. Based on the mass spectrometric analysis, 698 proteins were selected with a minimal fold change value of 2 and a significant difference between RasGEF_Flag_‐containing and the control lysate (*P* < 0.05). Main functions were determined by GO (Gene Ontology) analysis; the diagram shows fold enrichment for the indicated categories. (D) RasGEF_Flag_ specifically interacts with RAS2P *in vitro*. Schematic outline of the experiment. GST‐tag expressed in *E. coli* was used as a negative control. A representative western blot is shown (*n* = 2). The data were presented as mean ± SEM. Statistical analysis was performed using repeated measures ANOVA followed by Tukey's LSD test. ns, not significant; **P < 0.005.

RAS‐mediated signaling is coupled to the cAMP/PKA pathway in fungi which in turn is known to respond to changes in glucose levels [28, 47, 48, 49, 50]. Hence, we compared cAMP levels in *Δrasgef* and *wt* in response to glucose. While cAMP generation was significantly induced by glucose in *wt*, no changes in cAMP levels could be detected in *Δrasgef* (Fig. 4A), indicating that RasGEF activity is coupled to glucose‐dependent cAMP signaling in *Neurospora*.

To gain further insight into the function of RasGEF, we searched for interacting proteins in a pull‐down experiment. The protein lysate of the *rasgef*_*Flag*_ was subjected to affinity purification using an anti‐FLAG antibody. *wt* (FGSC #2489) lysates served as control. Following mass spectrometry analysis, RasGEF_Flag_‐associated proteins were identified and quantified. Among monomeric G proteins, RAS2P was selected based on the highest fold change (FC) and the lowest false discovery rate (FDR) values (Fig. 4B, Table S1). Gene ontology overrepresentation analysis based on molecular function revealed significant enrichment of proteins linked to GTPase activator function, indicating that the putative RasGEF functions in signaling routes involving monomeric G proteins (Fig. 4C).

To confirm the interaction between RasGEF and RAS2P, we expressed a GST‐RAS2P fusion protein in *E. coli* and used it as a bait for a pull‐down experiment. The resin specifically bound RasGEF_Flag_ (Fig. 4D), further indicating that RAS2P is an interactor of RasGEF.

### Adaptation of the circadian clock to glucose availability depends on RasGEF‐mediated signaling in mammalian cells

**Fig. 5:**
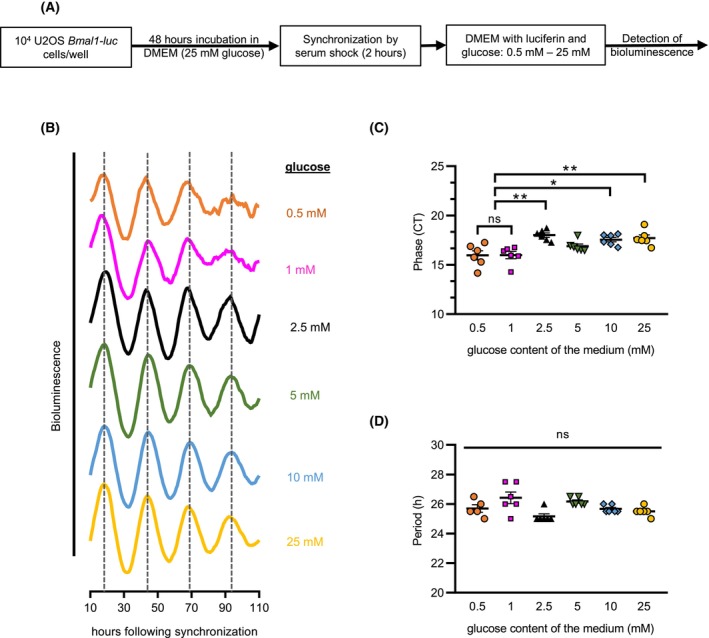
Nutritional compensation of the mammalian circadian clock in a wide range of nutrient levels. (A) Schematic outline of the experiment for the detection of *Bmal1* promoter activity in U2OS cells at various glucose levels. (B) Rhythmic *Bmal1* promoter activity can be detected in a wide range of glucose levels. Following synchronization, *Bmal1‐luc* cells were cultured in the presence of the indicated glucose concentration. Luminescence was detected for 5 days following synchronization. For better comparison, luminescence signals were detrended and normalized to the moving average. Averaged traces are shown (*n* = 5–6). To avoid overlaps and for better visualization, luminescence traces were shifted. (C) Effect of glucose levels on the phase of *bmal1* promoter activity in U2OS cells. Acrophases in circadian time (CT) from the experiment described in (B) are shown (*n* = 6). (D) Glucose availability does not affect the circadian period in U2OS cells. Averaged periods from the experiment described in (B) are indicated (*n* = 5–6). The data were presented as mean (B) or mean ± SEM (C, D). Statistical analysis was performed using one‐way ANOVA followed by Tukey's HSD test (C, D). ns, not significant; *P < 0.05; **P < 0.005.

To examine the conserved nature of our findings, we used human osteosarcoma cells (U2OS) expressing luciferase under the control of the *Bmal1* promoter (*Bmal1‐luc*) (Fig. 5A) [51] and supplemented the medium with various amounts of glucose. As differences in glucose levels lead to substantial differences in cell number that affect signal intensity, luminescence traces were normalized to the moving average. Between 25 mm (standard glucose concentration of culture media) and 0.5 mm glucose levels, *Bmal1‐luc* was rhythmically expressed (Fig. 5B). With decreasing glucose concentration, the amplitude (peak : trough) of the rhythm decreased and a slight phase advance was detected (Fig. 5C); however, the period of the oscillation remained constant (Fig. 5D). The data suggest metabolic compensation of the clock function over a wide range of glucose concentration.

**Fig. 6:**
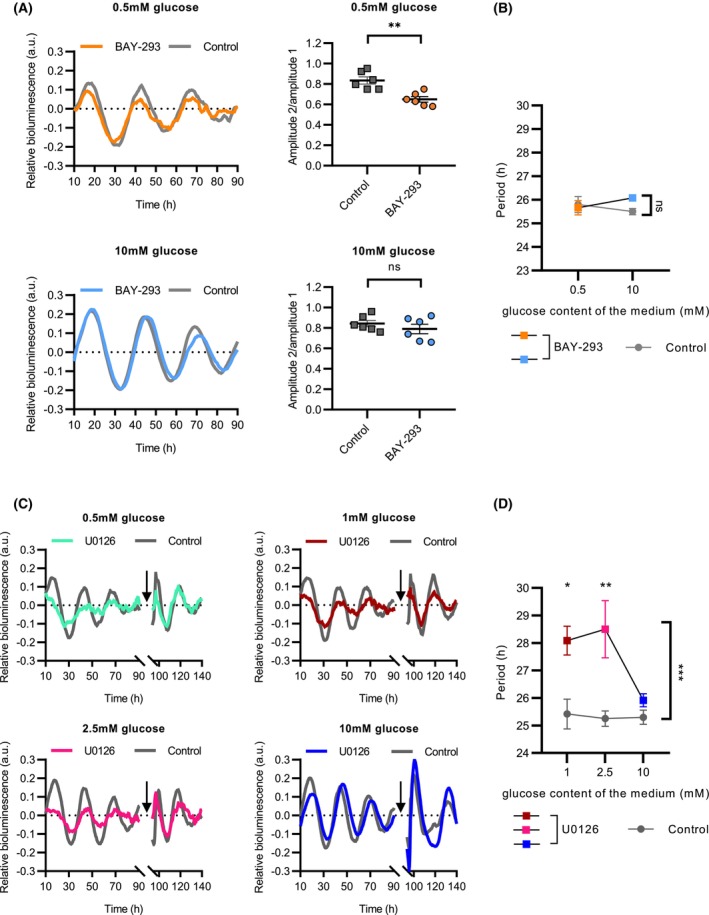
A RasGEF‐mediated pathway affects the metabolic compensation of the mammalian circadian clock. (A) Inhibition of SOS1 results in rhythm dampening at low glucose level. The experiment was performed as described in Fig. 5. Following synchronization, the cell culture medium was supplemented with either the SOS1 inhibitor BAY‐293 (600 nm) or vehicle (DMSO), as indicated. Left panels: luminescence signals were detrended and normalized to the moving average (indicator of cell number). Averaged traces are shown (*n* = 6). Right panels: Amplitude changes from the first to the second day in the presence or absence of BAY‐293 at the indicated glucose levels. Amplitude ratios (difference in the normalized bioluminescence between the second peak and nadir/difference in the normalized bioluminescence between the first peak and nadir) were calculated (*n* = 6). (B) Inhibition of SOS1 does not affect the circadian period in U2OS cells. Averaged periods based on data from the experiment described in (A) are indicated (*n* = 6). The color of each point in the diagram corresponds to the condition represented by the same color in (A). (C) Inhibition of the ERK pathway significantly reduces the rhythmic activity of the *Bmal1* promoter under low glucose conditions. The experiment was performed as described in Fig. 5A,B. Following synchronization, the cell culture medium was supplemented with either the ERK inhibitor U0126 (2 μm) or vehicle (DMSO), as indicated. After 90 h, the inhibitor was washed out and the cells were resuspended in fresh medium containing the original glucose levels (0.5 mm – 10 mm). The black arrows indicate the time point of medium change. Luminescence signals were detrended and normalized to the moving average (indicator of cell number) (*n* = 6). (D) Metabolic compensation of the circadian period is impaired in the presence of the ERK inhibitor. Averaged periods from the experiment described in (C) are indicated at various glucose levels (*n* = 6). Due to the irregularity of the rhythm in the inhibitor‐treated cells, a circadian period could not be determined at 0.5 mm glucose concentration. The color of each point in the diagram corresponds to the condition represented by the same color in (C). The data were presented as mean (A—left part, C) or mean ± SEM (A—right part, B, D). Statistical analysis was performed using two‐way ANOVA followed by Fisher's LSD test (A) and by Tukey's HSD test (B, D), which revealed significant group effect (D). ns, not significant; *P < 0.05; **P < 0.005; ***P < 0.001.

To address whether SOS1, the mammalian homolog of the *Neurospora* RasGEF, is involved in the maintenance of clock function under low glucose concentration, we treated the cells with a specific SOS1 inhibitor, BAY‐293 [52] (Fig. 6A). At low glucose level (0.5 mm), *Bmal1*‐luciferase oscillation in the inhibitor‐treated cells exhibited lower amplitude and dampened more quickly compared to nontreated control cells, while the rhythm was not affected by BAY‐293 at higher glucose concentration (10 mm) (Fig. 6A). Due to the weakening of the oscillation, the period could only be calculated based on data of 2–3 days; however, there was no significant difference (Fig. 6B). SOS1 regulates the RAS/MAPK/ERK pathway, which in turn can modulate the circadian clock [53]. To investigate how inhibition of this pathway affects clock oscillation at different glucose levels, we employed U0126, which inhibits the ERK activators MEK1/2, thereby suppressing ERK phosphorylation and signaling. U0126 flattened the oscillation and lengthened the period at low glucose concentrations (Fig. 6C,D). However, at high glucose level (10 mm), the luciferase signals were similar in the presence and absence of the inhibitor, and no period difference was obtained. After 90 h, the inhibitor was washed out and the cells were resuspended in fresh media containing the original glucose levels. The oscillations quickly recovered, indicating that the amplitude flattening was indeed a result of clock function inhibition rather than differences in cell number.

In summary, our results suggest that both SOS1 and ERK are involved in the control of the mammalian circadian clock at low glucose levels.

## Discussion

Metabolic compensation is a prerequisite of robust endogenous time measuring in an environment where nutrient availability shows significant variations. Accurate time‐of‐the‐day‐dependent adaptation of metabolic processes to cycling environmental conditions, such as light–dark or temperature changes, might be particularly critical when nutrient supply is significantly limited. We recently showed that the circadian TTFL of *Neurospora* functions with substantially altered levels and stoichiometry of its key elements during long‐term glucose starvation [31]. Results of our present study suggest that RasGEF‐mediated signaling, the activity of which is affected by metabolic conditions, supports robust function of the circadian clock under low glucose conditions in both fungi and mammalian cells. Involvement of RAS GTPases in regulating the circadian clock has been suggested across various levels of phylogeny [54, 55, 56, 57, 58, 59, 60]; however, their role in sustaining rhythmic tissue function under nutrient scarcity has not been previously described.

In *Neurospora*, the entrained phase of conidiation was delayed and both metabolic and temperature compensation of the circadian period were impaired on minimal medium in *Δrasgef* compared to *wt*. The altered response of the mutant to starvation could be followed at the level of the molecular oscillator as well; the mesor of *frq* expression was elevated and its rhythm was reduced compared to *wt* when glucose was depleted. The increased *frq* levels under starvation conditions were in accordance with the higher expression of WCC in *Δrasgef* relative to *wt*. Changes in the clock output could also be followed at the level of gene expression. Both the upregulation and the oscillation of *ccg‐2* were impaired in *Δrasgef* under starvation conditions, suggesting an important role of RasGEF in adapting the reproductive strategy of *Neurospora* to the nutrient status and thus enhancing the ecological fitness of the fungus.

Expression of *rasgef* showed circadian variations. *rasgef* RNA peaked in the subjective night, and in accordance with this, was slightly repressed by light. The *wt* phase and period of conidiation did not depend on the endogenous rhythm of *rasgef*, since the mutant phenotype could be rescued by *ccg‐1* promoter‐driven expression of *rasgef*. Similar to its yeast homolog CDC25 [44], *Neurospora* RasGEF was detected in both the cytosolic and the nuclear fractions and displayed glucose‐dependent phosphorylation and localization. In the presence of glucose, phosphorylation of CDC25 is dependent on PKA activity and is thought to be part of a negative feedback regulation within the CDC25/RAS/cAMP/PKA pathway, leading to dissociation of RAS from CDC25 [45, 46]. In addition, enhanced PKA activity which might be coupled to high glucose conditions induces nuclear export of phosphorylated CDC25 [37]. This observation is in accordance with our data indicating that hypophosphorylated RasGEF accumulates in the nuclear fraction of starving *Neurospora*. Besides influencing posttranslational modification and subcellular localization, glucose also impacted the RNA levels of RasGEF, leading to increased expression under standard conditions compared to starvation.

The impaired cAMP response of *Δrasgef* to glucose suggests that RasGEF activates a RAS/cAMP/PKA pathway. PKA acting both directly and indirectly on clock components is a modulator and fine‐tuner of clock function in *Neurospora*. It is a priming kinase for subsequent phosphorylation of the WCC by CK1a and/or CK2, contributing to the inactivation of the transcription factor [61]. PKA also interacts with FRQ and enhances its stability, and it may indirectly affect the molecular clock by increasing the translational activity of the cell [30] and activating PP2A [62], which can dephosphorylate both the WCC and FRQ [21, 36, 63, 64].

In the genome of *Saccharomyces cerevisiae*, only two *ras* genes, *ras1* and *ras2*, can be identified, whereas the *Neurospora crassa* genome possesses three *ras* genes, *ras1*, *ras2*, and *ras2p*. Both our mass spectrometry analysis and *in vitro* interaction assay indicated that RAS2P was the primary monomeric G protein partner of *Neurospora* RasGEF. To our knowledge, RAS2P has not been previously characterized in *Neurospora*. It is a 317 amino acid protein sharing a sequence identity of 37% and similarity of 43% with *Neurospora* RAS2, respectively [65]. Homologs of RAS2P are present in other fungi; however, we have not found any literature data providing information about their specific functions. It might be important to note that in some articles about *Saccharomyces cerevisiae*, RAS2 and RAS2P are used synonymously [46, 66], meaning they refer to the same protein encoded by the *ras2* gene, the closest homolog of which is *ras2* in *Neurospora*. An interaction between RAS2 and RasGEF was not expected, as RasGEF‐dependent signaling and RAS2 seem to control the nutritional compensation of the *Neurospora* circadian clock through distinct mechanisms: the *ras2* deletion strain exhibits a pronounced circadian phenotype under high glucose conditions [28], while the RasGEF/RAS2P pathway appears to be necessary for maintaining clock function during glucose starvation. In the last 13 years, several factors have been identified that participate in the metabolic compensation of the *Neurospora* clock [25, 26, 27, 28, 29, 30, 31], and this makes it clear that only a highly complex regulation can stabilize the clock's speed despite fluctuations in nutrient availability. Further investigations may reveal the mechanism how RAS2P is inserted into this network.

Period and amplitude of the oscillation of *Bmal1*‐promoter activity was earlier demonstrated to be sensitive to glucose availability in U2OS cells [67]. However, Kelliher *et al*. [25] recently showed metabolic compensation of the circadian period when cells were cultured in the presence of either 5.5 or 25 mm glucose. Our data suggest that circadian time measuring is metabolically compensated within an even larger range of glucose concentrations (0.5–25 mm) and its robustness is dependent on SOS1 and ERK.

In humans, blood glucose concentration below 2.8 mm poses a life‐threatening condition; however, interstitial glucose levels might be largely different from the plasma levels, dependent on the type and metabolic activity of the tissue [68, 69, 70]. Additionally, glucose concentrations around the cells can differ based on their distance from the capillaries and fluctuations in local blood supply. Mechanisms that sustain cellular clock function during shorter or longer periods of interstitial glucose reductions, including ischemic episodes caused by pathological circulatory changes, can ensure that endogenous timekeeping remains synchronized at both the tissue and organism levels, which is essential for successful adaptation. Recently, factors regulating gene expression and mRNA stability were identified as influencing nutritional compensation in both *Neurospora* and mammalian cells [25]. While these mechanisms may rather contribute to long‐term stabilization of the circadian period, the RasGEF/SOS1 pathway could also support rapid compensatory responses [71] of the circadian clock to fluctuations of glucose levels. The exact mechanisms by which the SOS1‐mediated pathway in mammalian cells and the RasGEF signaling in *Neurospora* support clock function under starvation conditions remain to be elucidated in future studies.

While mammalian RAS proteins are mostly studied as oncogenic factors and activating RAS mutations are hallmarks of certain cancer types, SOS1/RAS/ERK‐dependent signaling is also vital for regulating the development, growth, and regeneration of nontumorous tissues [72]. In addition, prevention of cell death under glucose‐deprived conditions is also dependent on intact SOS function [73]. In MEFs lacking SOS1, levels of lactate dehydrogenase A become reduced, and kinetics of phospho‐AMP‐activated protein kinase signaling is delayed following glucose withdrawal when compared to the response of the control cells. Moreover, in SOS1/SOS2 double KO MEFs, cleaved caspase‐3 can be detected in starved cells, indicating an apoptotic response to glucose deprivation. The understanding of how glucose supply can affect RasGEF function is still incomplete. Recent data suggest an evolutionary conserved mechanism in which fructose‐1,6‐bisphosphate derived from glycolysis directly regulates CDC25 and SOS1 in yeast and mammalian cells, respectively [74]. Activation of MAPK pathways was also demonstrated under nutrient‐depleted conditions in both fungi and mammals, where these pathways induce autophagy to support survival [75, 76].

Taken together, our data indicate compensation of the circadian period across a broad range of glucose concentrations in both *Neurospora* and human cells, and uncover the conserved role of RasGEF activity in maintaining endogenous time measurement under low glucose conditions.

## Materials and methods

### Plasmid construction

To construct the *pMF‐ccg1‐2xFlag‐rasgef* plasmid, the coding sequence of *rasgef* was amplified using *wt* cDNA as a template and the forward primer 5′‐AAAAAGGCGCGCCGCATCGCAGAGTAGCCGAC‐3′ and the reverse primer 5′‐AAAAACCCGGGCTAGACTTGAGCAGAGGTAGGCA‐3′. The PCR product was inserted as an AscI‐XmaI fragment into the *pMF‐ccg1‐2xFlag‐frh* vector [77] to replace the *frh* sequence. To assemble *pBM61‐rasgef‐luc*, a 1493 bp long 5′ untranslated region of the *rasgef* gene was amplified using the forward primer 5′‐AAAAGGATCCGACCTACCTACCTCCAATATC‐3′ and the reverse primer 5′‐AAAAGCGGCCGCGGCTGTCGGATGCGAGAAG‐3′ and gDNA as a template. A BamHI‐NotI fragment of the PCR product was used to replace the *frq* promoter in the *pBM60‐Pfrq‐luc‐trpC* vector [78]. *pGEX4‐ras2p* was constructed by inserting the coding region of *ras2p* between the EcoRI and NotI sites of the *pGEX4T1* (Cytiva, Marlborough, Massachusetts, United States) vector. The coding region of *ras2p* was amplified using the forward primer 5′‐AAAAAGAATTCATGCCATCCCAAAATCCCCGTAG‐3′ and the reverse primer 5′‐AGCGGCCGCTTACCAGCACCTCAACGACTTCAAC‐3′ and *wt* cDNA as template.

### *Neurospora* strains and culture conditions

*Neurospora crassa* strains *wt* (FGSC #2489), *wt*,*bd* (FGSC #1858), and *Δrasgef* (FGSC #11866) were obtained from the Fungal Genetics Stock Center (Kansas City, Kansas, USA) [79]. *wt*‐*pfrq‐luc* was generated in a previous project [33]. FGSC #11866 was created during the *Neurospora* Genome Project [36]. *Δrasgef*, *bd* and *his3*, *Δrasgef* were generated by crossing *Δrasgef* with *wt*,*bd* or *wt*, *his3* (FGSC #6103), respectively. Generation of *his3*, *Δrasgef*, *bd* was carried out by crossing *Δrasgef*, *bd* with *his3*, *bd* (87–74). To generate *rasgef*_Flag_ and *rasgef*_Flag_, *bd*, *pMF‐ccg1‐2xFlag‐rasgef was* used for transformation of *his3*, *Δrasgef* and *his3*, *Δrasgef*, *bd*, respectively [80]. To create the *wt‐rasgef‐luc* strain, *wt*, *his3* was transformed with *pBM61‐rasgef‐luc*.

The solid slant medium contained Vogel's medium [81], supplemented with 50 ng·mL^−1^ biotin, 2% glucose, and 2% agar. The standard liquid medium contained Vogel's medium supplemented with 0.5% l‐arginine, 10 ng·mL^−1^ biotin, and 2% glucose. In the starvation medium (also referred to as the low glucose condition), the glucose concentration was reduced to 0.01%. Liquid cultures were inoculated with mycelial mats formerly grown in Petri dishes. Importantly, at least 150 mL of liquid medium per mycelial discs was used in order to keep the glucose concentration as constant as possible during the incubation.

In the minimal race tube medium, glucose was omitted, 0.17% arginine and 3.2% agar were added [28, 31, 33, 82]. Race tubes (320 × 15 mm) were inoculated with conidia and incubated at 25 °C when otherwise not indicated. The growth front was marked at specific time points and either the circadian phase or period was analyzed using the chronosx 2.0.3.4 software (developed by T. Roenneberg) according to the protocol described previously [33].

### RNA analysis

Total RNA was extracted using the Tri Reagent (Sigma‐Aldrich, Merck KGaA, Darmstadt, Germany) isolation reagent, and transcript levels were quantified as described earlier [33]. Values were normalized to the *wt* grown in 2% glucose‐containing standard medium in each experiment, unless indicated otherwise. *gna‐3* was used as a housekeeping gene [31].

### Protein analysis

Extraction of *Neurospora* protein, western blots [33, 64] and subcellular fractionation [28, 83] were performed as described earlier. In subcellular fractionation, the same protein amounts of cytosolic and nuclear fractions were analyzed. Ponceau S staining is shown as loading control (LC). Representative western blots are shown. Where indicated, protein extracts were prepared without phosphatase inhibitors and treated with λ phosphatase (Invitrogen, Carlsbad, CA, USA) (2 U·mg^−1^ protein) for 60 min at 30 °C.

### Detection of cAMP levels

*Neurospora* total cell lysates were used for the cAMP assay, and instructions of the manufacturer were followed (cAMP‐Screen Direct™ Cyclic AMP Immunoassay System, Applied Biosystems, Waltham, MA, USA).

### Expression of GST‐RAS2P and investigation of its interaction with RasGEF

Rosetta™(DE3) Competent Cells (Novagen, Merck KGaA, Darmstadt, Germany) were transformed with *pGEX4 or pGEX4‐ras2p* according to the manufacturer's recommendations. Expression of the fusion protein was induced with 0.1 mm isopropyl‐beta‐d‐thiogalactopyranoside for 2 h at 37 °C followed by an overnight incubation at 15 °C. Following the manufacturer's recommendations, rosetta cells were harvested and lysed. GST or GST‐RAS2P were bound to the glutathione‐agarose beads (GE Healthcare Life Sciences, Chicago, IL, UStates). The beads were washed with TBS, followed by incubation with 20 mg of *rasgef*_Flag_ lysates in a total volume of 1.5 mL. Samples were incubated under agitation for 1.5 h at 4 °C. Beads were washed three times with 1.5 mL TBS, and proteins were eluted with SDS‐sample buffer.

### *In vivo* luciferase assays in *Neurospora crassa*

96‐well white plates with solid medium containing Vogel's salt solution were inoculated with the luciferase‐expressing strains [33]. Cultures were incubated for 2 days in constant light at 30 °C. After the light/dark transfer, luminescence was detected with a Polarstar Optima luminometer (BMG Labtech, Ortenberg, Germany) using an integration time of 4 s. Data analysis and interpretation were performed as described [33].

### Mass spectrometry analysis

*wt* (FGSC #2489) and *rasgef*_Flag_ (*bd* ^*−*^) grown in LL (constant light) were harvested, frozen in liquid nitrogen, and ground with TissueLyser (Qiagen, Hilden, Germany) (50 Hz, 120 s). Total proteins were extracted as described [84] and immunopurified (4 mg/IP) using anti‐FLAG antibody coupled to 50 nm size magnetic beads (MACS® Technology, Miltenyi Biotec, Bergisch Gladbach, Germany) with a method modified [85, 86]. The further steps of sample preparation and the mass spectrometry analysis were performed as previously described [87]. Briefly, the samples were reduced with dithiothreitol (10 mm), alkylated by iodoacetamide (22 mm), and digested in column with trypsin (Promega, Madison, Wisconsin, United States). The digestion mixtures were acidified and transferred to a single‐use trapping mini‐column (Evotip; 1/8 of the samples) and then analyzed with a data‐dependent LC–MS/MS method using an Evosep One (LC: 15 SPD; MS1: R:120000) on‐line coupled to a linear ion‐trap‐Orbitrap (Orbitrap‐Fusion Lumos, Thermo Fisher Scientific, Waltham, MA, USA) mass spectrometer operating in positive ion mode. Data acquisition was carried out in a data‐dependent fashion; multiply charged ions were selected in cycle time from each MS survey scan for ion‐trap HCD fragmentation (MS spectra were acquired in the Orbitrap (*R* = 60 000) while MSMS in the ion‐trap).

### Interpretation of mass spectrometry data

Analyzes and interpretation of mass spectrometry data were performed as described [87, 88]. Briefly, raw data were converted into peak lists using the in‐house Proteome Discoverer (v1.4) and searched against the UniProt NEUCR database and 6 user defined proteins (downloaded 2022.07.20, 23 795 proteins) using our in‐cloud Protein Prospector search engine (v5.15.1) with the following parameters: enzyme: trypsin with maximum 2 missed cleavages; mass accuracies: 5 ppm for precursor ions and 0.6 Da for fragment ions (both monoisotopic); fixed modification: carbamidomethylation of Cys residues; variable modifications: acetylation of protein N‐termini; Met oxidation; cyclization of N‐terminal Gln residues, allowing maximum 2 variable modifications per peptide. Acceptance criteria: minimum scores: 22 and 15; maximum E values: 0.01 and 0.05 for protein and peptide identifications, respectively.

13 IPs (5 *wt*, 8 *rasgef*_*Flag*_) were used for meta‐analyses. The statistical analyses were performed by edgeR [89] using a modified spectral counting (PSMxCoverage %) [90] to determine the relative abundance of individual proteins (Label free quantitation) [86]. We used a cutoff of *P* value 0.05, and a fold change (FC) relative to the negative controls was set to 1.5, with the restrictions that the minimum detected Unique peptides in the samples and also the coverage % must be higher than in the highest detected value in the negative controls. We accepted protein identifications if at least 3 unique peptides were annotated.

### *In vivo* luciferase assays in U2OS cells

10^4^ U2OS *Bmal1‐luc* cells/well were inoculated in a 96‐well plate 48 h before the measurement [51]. Synchronization of the cells was performed by serum‐shock using 20% FBS containing DMEM solution for 2 h followed by a medium change to DMEM (w/o Phenol red and glucose) containing 10% FBS, 50 mm Hepes (pH = 7.4), 1% penicillin/streptomycin, 1% l‐glutamine, 200 μm luciferin, and glucose as indicated. Plates were sealed by optic foils and bioluminescence was recorded every 30 min at 37 °C for 5–6 days by an Enspire Multimode Plate Reader (PerkinElmer, Waltham, MA, USA). To remove U0126, cells were washed twice with phosphate‐buffered saline, resynchronized, and resuspended in the original medium without inhibitor.

### Statistical analysis

For statistical analysis, the Statistica 13 (Statsoft Inc., Tulsa, OK, USA) software was used. Error bars indicate ±SEM. Results were considered statistically significant when the *P* value was <0.05 (*P < 0.05; **P < 0.01; ***P < 0.001; n.s.: nonsignificant). Cosinor analysis was performed with GraphPad Prism 8.0, R 4.3.3, and R studio (version 2024.04.2 + 764). Further statistical details can be found in the figure legends. *n* represents the number of independent biological samples.

## Conflict of interest

The authors declare no competing interests.

## Author contributions

Conceptualization, KK and NG; investigation and analysis, OS, AS, EÉK, and AP‐S; resources and funding acquisition, KK, NG and MB; data curation, OS, KK, AP‐S, and AS; writing and editing, OS, MB and KK.

## Supporting information


**Fig. S1.** The growth and circadian phenotype of *Δrasgef*.
**Table S1.** List of the proteins with RasGEF_Flag_‐specific enrichment according to the criteria detailed in the Materials and Methods section.

## Data Availability

The mass spectrometry proteomics data have been deposited to the ProteomeXchange Consortium via the PRIDE [91] partner repository with the dataset identifier PXD062726. All other datasets used in this study are available from the corresponding author on request.

## References

[1] Dodd AN , Salathia N , Hall A , Kevei E , Toth R , Nagy F , Hibberd JM , Millar AJ & Webb AA (2005) Plant circadian clocks increase photosynthesis, growth, survival, and competitive advantage. Science 309, 630–633. doi: 10.1126/science.1115581 16040710

[2] Hozer C , Perret M , Pavard S & Pifferi F (2020) Survival is reduced when endogenous period deviates from 24 h in a non‐human primate, supporting the circadian resonance theory. Sci Rep 10, 18002. 33093578 10.1038/s41598-020-75068-8 PMC7582969

[3] Ouyang Y , Andersson CR , Kondo T , Golden SS & Johnson CH (1998) Resonating circadian clocks enhance fitness in cyanobacteria. Proc Natl Acad Sci U S A 95, 8660–8664. 9671734 10.1073/pnas.95.15.8660 PMC21132

[4] Spoelstra K , Wikelski M , Daan S , Loudon AS & Hau M (2016) Natural selection against a circadian clock gene mutation in mice. Proc Natl Acad Sci U S A 113, 686–691. doi: 10.1073/pnas.1516442113 26715747 PMC4725470

[5] Woelfle MA , Ouyang Y , Phanvijhitsiri K & Johnson CH (2004) The adaptive value of circadian clocks: an experimental assessment in cyanobacteria. Curr Biol 14, 1481–1486. doi: 10.1016/j.cub.2004.08.023 15324665

[6] Fishbein AB , Knutson KL & Zee PC (2021) Circadian disruption and human health. J Clin Invest 131, e148286. 34596053 10.1172/JCI148286 PMC8483747

[7] Lee Y (2021) Roles of circadian clocks in cancer pathogenesis and treatment. Exp Mol Med 53, 1529–1538. 34615982 10.1038/s12276-021-00681-0 PMC8568965

[8] Diernfellner ACR & Brunner M (2020) Phosphorylation timers in the *Neurospora crassa* circadian clock. J Mol Biol 432, 3449–3465. 32305463 10.1016/j.jmb.2020.04.004

[9] Dunlap JC & Loros JJ (2017) Making time: conservation of biological clocks from fungi to animals. Microbiol Spectr 5, 2016. 10.1128/microbiolspec.funk-0039-2016 PMC5446046 28527179

[10] Gallego M & Virshup DM (2007) Post‐translational modifications regulate the ticking of the circadian clock. Nat Rev Mol Cell Biol 8, 139–148. 17245414 10.1038/nrm2106

[11] Hogenesch JB & Ueda HR (2011) Understanding systems‐level properties: timely stories from the study of clocks. Nat Rev Genet 12, 407–416. 21556016 10.1038/nrg2972

[12] Partch C & Brunner M (2022) How circadian clocks keep time: the discovery of slowness. FEBS Lett 596, 1613–1614. 35775878 10.1002/1873-3468.14432

[13] Rosbash M (2009) The implications of multiple circadian clock origins. PLoS Biol 7, e62. 19296723 10.1371/journal.pbio.1000062 PMC2656552

[14] Takahashi JS (2017) Transcriptional architecture of the mammalian circadian clock. Nat Rev Genet 18, 164–179. 27990019 10.1038/nrg.2016.150 PMC5501165

[15] Cheng P , He Q , He Q , Wang L & Liu Y (2005) Regulation of the Neurospora circadian clock by an RNA helicase. Genes Dev 19, 234–241. 15625191 10.1101/gad.1266805 PMC545885

[16] Gorl M , Merrow M , Huttner B , Johnson J , Roenneberg T & Brunner M (2001) A PEST‐like element in FREQUENCY determines the length of the circadian period in *Neurospora crassa* . EMBO J 20, 7074–7084. 11742984 10.1093/emboj/20.24.7074 PMC125781

[17] Schafmeier T , Haase A , Kaldi K , Scholz J , Fuchs M & Brunner M (2005) Transcriptional feedback of Neurospora circadian clock gene by phosphorylation‐dependent inactivation of its transcription factor. Cell 122, 235–246. 16051148 10.1016/j.cell.2005.05.032

[18] Larrondo LF , Olivares‐Yanez C , Baker CL , Loros JJ & Dunlap JC (2015) Circadian rhythms. Decoupling circadian clock protein turnover from circadian period determination. Science 347, 1257277. doi: 10.1126/science.1257277 25635104 PMC4432837

[19] Tang CT , Li S , Long C , Cha J , Huang G , Li L , Chen S & Liu Y (2009) Setting the pace of the Neurospora circadian clock by multiple independent FRQ phosphorylation events. Proc Natl Acad Sci U S A 106, 10722–10727. 19506251 10.1073/pnas.0904898106 PMC2705601

[20] Wang B , Stevenson EL & Dunlap JC (2023) Functional analysis of 110 phosphorylation sites on the circadian clock protein FRQ identifies clusters determining period length and temperature compensation. G3 13, jkac334. doi: 10.1093/g3journal/jkac334 36537198 PMC9911066

[21] Gyongyosi N & Kaldi K (2014) Interconnections of reactive oxygen species homeostasis and circadian rhythm in *Neurospora crassa* . Antioxid Redox Signal 20, 3007–3023. 23964982 10.1089/ars.2013.5558

[22] Froehlich AC , Liu Y , Loros JJ & Dunlap JC (2002) White Collar‐1, a circadian blue light photoreceptor, binding to the frequency promoter. Science 297, 815–819. 12098706 10.1126/science.1073681

[23] He Q , Cheng P , Yang Y , Wang L , Gardner KH & Liu Y (2002) White collar‐1, a DNA binding transcription factor and a light sensor. Science 297, 840–843. 12098705 10.1126/science.1072795

[24] Ella K , Mocsai A & Kaldi K (2018) Circadian regulation of neutrophils: control by a cell‐autonomous clock or systemic factors? Eur J Clin Invest 48(Suppl 2), e12965. 29877596 10.1111/eci.12965

[25] Kelliher CM , Stevenson EL , Loros JJ & Dunlap JC (2023) Nutritional compensation of the circadian clock is a conserved process influenced by gene expression regulation and mRNA stability. PLoS Biol 21, e3001961. 36603054 10.1371/journal.pbio.3001961 PMC9848017

[26] Adhvaryu K , Firoozi G , Motavaze K & Lakin‐Thomas P (2016) PRD‐1, a component of the circadian system of *Neurospora crassa*, is a member of the DEAD‐box RNA helicase family. J Biol Rhythms 31, 258–271. doi: 10.1177/0748730416639717 27029286

[27] Emerson JM , Bartholomai BM , Ringelberg CS , Baker SE , Loros JJ & Dunlap JC (2015) Period‐1 encodes an ATP‐dependent RNA helicase that influences nutritional compensation of the Neurospora circadian clock. Proc Natl Acad Sci U S A 112, 15707–15712. 26647184 10.1073/pnas.1521918112 PMC4697410

[28] Gyongyosi N , Szoke A , Ella K & Kaldi K (2017) The small G protein RAS2 is involved in the metabolic compensation of the circadian clock in the circadian model *Neurospora crassa* . J Biol Chem 292, 14929–14939. 28729421 10.1074/jbc.M117.804922 PMC5592671

[29] Olivares‐Yanez C , Emerson J , Kettenbach A , Loros JJ , Dunlap JC & Larrondo LF (2016) Modulation of circadian gene expression and metabolic compensation by the RCO‐1 corepressor of *Neurospora crassa* . Genetics 204, 163–176. 27449058 10.1534/genetics.116.191064 PMC5012383

[30] Sancar G , Sancar C & Brunner M (2012) Metabolic compensation of the Neurospora clock by a glucose‐dependent feedback of the circadian repressor CSP1 on the core oscillator. Genes Dev 26, 2435–2442. 23124067 10.1101/gad.199547.112 PMC3490001

[31] Szoke A , Sarkany O , Schermann G , Kapuy O , Diernfellner ACR , Brunner M , Gyongyosi N & Kaldi K (2023) Adaptation to glucose starvation is associated with molecular reorganization of the circadian clock in *Neurospora crassa* . Elife 12, e79765. 36625037 10.7554/eLife.79765 PMC9831608

[32] Belden WJ , Larrondo LF , Froehlich AC , Shi M , Chen CH , Loros JJ & Dunlap JC (2007) The band mutation in *Neurospora crassa* is a dominant allele of RAS‐1 implicating RAS signaling in circadian output. Genes Dev 21, 1494–1505. 17575051 10.1101/gad.1551707 PMC1891427

[33] Gyongyosi N , Nagy D , Makara K , Ella K & Kaldi K (2013) Reactive oxygen species can modulate circadian phase and period in *Neurospora crassa* . Free Radic Biol Med 58, 134–143. 23277144 10.1016/j.freeradbiomed.2012.12.016

[34] UniProt C (2025) UniProt: the universal protein knowledgebase in 2025. Nucleic Acids Res 53(D1), D609–D617. doi: 10.1093/nar/gkae1010 39552041 PMC11701636

[35] Santangelo GM (2006) Glucose signaling in *Saccharomyces cerevisiae* . Microbiol Mol Biol Rev 70, 253–282. 16524925 10.1128/MMBR.70.1.253-282.2006 PMC1393250

[36] Colot HV , Park G , Turner GE , Ringelberg C , Crew CM , Litvinkova L , Weiss RL , Borkovich KA & Dunlap JC (2006) A high‐throughput gene knockout procedure for Neurospora reveals functions for multiple transcription factors. Proc Natl Acad Sci U S A 103, 10352–10357. 16801547 10.1073/pnas.0601456103 PMC1482798

[37] Belotti F , Tisi R , Paiardi C , Groppi S & Martegani E (2011) PKA‐dependent regulation of Cdc25 RasGEF localization in budding yeast. FEBS Lett 585, 3914–3920. 22036786 10.1016/j.febslet.2011.10.032

[38] Belotti F , Tisi R , Paiardi C , Rigamonti M , Groppi S & Martegani E (2012) Localization of Ras signaling complex in budding yeast. Biochim Biophys Acta 1823, 1208–1216. 22575457 10.1016/j.bbamcr.2012.04.016

[39] Quadri R , Galli M , Galati E , Rotondo G , Gallo GR , Panigada D , Plevani P & Muzi‐Falconi M (2020) Haspin regulates Ras localization to promote Cdc24‐driven mitotic depolarization. Cell Discov 6, 42. 32595981 10.1038/s41421-020-0170-2 PMC7308332

[40] Bell‐Pedersen D , Dunlap JC & Loros JJ (1992) The Neurospora circadian clock‐controlled gene, ccg‐2, is allelic to eas and encodes a fungal hydrophobin required for formation of the conidial rodlet layer. Genes Dev 6, 2382–2394. 1459460 10.1101/gad.6.12a.2382

[41] Kaldenhoff R & Russo VE (1993) Promoter analysis of the bli‐7/eas gene. Curr Genet 24, 394–399. doi: 10.1007/BF00351847 8299154

[42] Sokolovsky VY , Lauter FR , Mullerrober B , Ricci M , Schmidhauser TJ & Russo VEA (1992) Nitrogen regulation of blue light‐inducible genes in Neurospora‐Crassa. J Gen Microbiol 138, 2045–2049.

[43] Bell‐Pedersen D , Shinohara ML , Loros JJ & Dunlap JC (1996) Circadian clock‐controlled genes isolated from *Neurospora crassa* are late night‐ to early morning‐specific. Proc Natl Acad Sci U S A 93, 13096–13101. 8917550 10.1073/pnas.93.23.13096 PMC24052

[44] Tisi R , Belotti F , Paiardi C , Brunetti F & Martegani E (2008) The budding yeast RasGEF Cdc25 reveals an unexpected nuclear localization. Biochim Biophys Acta 1783, 2363–2374. 18930081 10.1016/j.bbamcr.2008.09.004

[45] Gross E , Goldberg D & Levitzki A (1992) Phosphorylation of the S. Cerevisiae Cdc25 in response to glucose results in its dissociation from Ras. Nature 360, 762–765. 1334534 10.1038/360762a0

[46] Jian D , Aili Z , Xiaojia B , Huansheng Z & Yun H (2010) Feedback regulation of Ras2 guanine nucleotide exchange factor (Ras2‐GEF) activity of Cdc25p by Cdc25p phosphorylation in the yeast *Saccharomyces cerevisiae* . FEBS Lett 584, 4745–4750. 21073870 10.1016/j.febslet.2010.11.006

[47] Lengeler KB , Davidson RC , D'Souza C , Harashima T , Shen WC , Wang P , Pan X , Waugh M & Heitman J (2000) Signal transduction cascades regulating fungal development and virulence. Microbiol Mol Biol Rev 64, 746–785. 11104818 10.1128/mmbr.64.4.746-785.2000 PMC99013

[48] Rolland F , De Winde JH , Lemaire K , Boles E , Thevelein JM & Winderickx J (2000) Glucose‐induced cAMP signalling in yeast requires both a G‐protein coupled receptor system for extracellular glucose detection and a separable hexose kinase‐dependent sensing process. Mol Microbiol 38, 348–358. 11069660 10.1046/j.1365-2958.2000.02125.x

[49] Van Zeebroeck G , Demuyser L , Zhang Z , Cottignie I & Thevelein JM (2020) Nutrient sensing and cAMP signaling in yeast: G‐protein coupled receptor versus transceptor activation of PKA. Microb Cell 8, 17–27. 33490229 10.15698/mic2021.01.740 PMC7780724

[50] Xiaojia B & Jian D (2010) Serine214 of Ras2p plays a role in the feedback regulation of the Ras‐cAMP pathway in the yeast *Saccharomyces cerevisiae* . FEBS Lett 584, 2333–2338. 20388513 10.1016/j.febslet.2010.04.011

[51] Maier B , Wendt S , Vanselow JT , Wallach T , Reischl S , Oehmke S , Schlosser A & Kramer A (2009) A large‐scale functional RNAi screen reveals a role for CK2 in the mammalian circadian clock. Genes Dev 23, 708–718. 19299560 10.1101/gad.512209 PMC2661607

[52] Hillig RC , Sautier B , Schroeder J , Moosmayer D , Hilpmann A , Stegmann CM , Werbeck ND , Briem H , Boemer U , Weiske J *et al*. (2019) Discovery of potent SOS1 inhibitors that block RAS activation via disruption of the RAS‐SOS1 interaction. Proc Natl Acad Sci U S A 116, 2551–2560. 30683722 10.1073/pnas.1812963116 PMC6377443

[53] Goldsmith CS & Bell‐Pedersen D (2013) Diverse roles for MAPK signaling in circadian clocks. Adv Genet 84, 1–39. 24262095 10.1016/B978-0-12-407703-4.00001-3 PMC4437509

[54] Bouchard‐Cannon P & Cheng HY (2012) Scheduled feeding alters the timing of the suprachiasmatic nucleus circadian clock in dexras1‐deficient mice. Chronobiol Int 29, 965–981. doi: 10.3109/07420528.2012.707264 22928915 PMC3707842

[55] Cheng HY , Dziema H , Papp J , Mathur DP , Koletar M , Ralph MR , Penninger JM & Obrietan K (2006) The molecular gatekeeper Dexras1 sculpts the photic responsiveness of the mammalian circadian clock. J Neurosci 26, 12984–12995. doi: 10.1523/JNEUROSCI.4253-06.2006 17167088 PMC6674968

[56] Cheng HY & Obrietan K (2006) Dexras1: shaping the responsiveness of the circadian clock. Semin Cell Dev Biol 17, 345–351. doi: 10.1016/j.semcdb.2006.03.006 16765612

[57] Koletar MM , Cheng HY , Penninger JM & Ralph MR (2011) Loss of dexras1 alters nonphotic circadian phase shifts and reveals a role for the intergeniculate leaflet (IGL) in gene‐targeted mice. Chronobiol Int 28, 553–562. doi: 10.3109/07420528.2011.592235 21834641

[58] Relogio A , Thomas P , Medina‐Perez P , Reischl S , Bervoets S , Gloc E , Riemer P , Mang‐Fatehi S , Maier B , Schafer R *et al*. (2014) Ras‐mediated deregulation of the circadian clock in cancer. PLoS Genet 10, e1004338. 24875049 10.1371/journal.pgen.1004338 PMC4038477

[59] Serchov T , Jilg A , Wolf CT , Radtke I , Stehle JH & Heumann R (2016) Ras activity oscillates in the mouse suprachiasmatic nucleus and modulates circadian clock dynamics. Mol Neurobiol 53, 1843–1855. 25762011 10.1007/s12035-015-9135-0

[60] Weber F , Hung HC , Maurer C & Kay SA (2006) Second messenger and Ras/MAPK signalling pathways regulate CLOCK/CYCLE‐dependent transcription. J Neurochem 98, 248–257. 16805811 10.1111/j.1471-4159.2006.03865.x

[61] Huang G , Chen S , Li S , Cha J , Long C , Li L , He Q & Liu Y (2007) Protein kinase a and casein kinases mediate sequential phosphorylation events in the circadian negative feedback loop. Genes Dev 21, 3283–3295. 18079175 10.1101/gad.1610207 PMC2113029

[62] Castermans D , Somers I , Kriel J , Louwet W , Wera S , Versele M , Janssens V & Thevelein JM (2012) Glucose‐induced posttranslational activation of protein phosphatases PP2A and PP1 in yeast. Cell Res 22, 1058–1077. 22290422 10.1038/cr.2012.20 PMC3367521

[63] Querfurth C , Diernfellner AC , Gin E , Malzahn E , Hofer T & Brunner M (2011) Circadian conformational change of the Neurospora clock protein FREQUENCY triggered by clustered hyperphosphorylation of a basic domain. Mol Cell 43, 713–722. doi: 10.1016/j.molcel.2011.06.033 21884974

[64] Schafmeier T , Kaldi K , Diernfellner A , Mohr C & Brunner M (2006) Phosphorylation‐dependent maturation of Neurospora circadian clock protein from a nuclear repressor toward a cytoplasmic activator. Genes Dev 20, 297–306. 16421276 10.1101/gad.360906 PMC1361701

[65] Camacho C , Coulouris G , Avagyan V , Ma N , Papadopoulos J , Bealer K & Madden TL (2009) BLAST+: architecture and applications. BMC Bioinformatics 10, 421. 20003500 10.1186/1471-2105-10-421 PMC2803857

[66] Dong J & Bai X (2011) The membrane localization of Ras2p and the association between Cdc25p and Ras2‐GTP are regulated by protein kinase a (PKA) in the yeast Saccharomyces cerevisiae. FEBS Lett 585, 1127–1134. 21457714 10.1016/j.febslet.2011.03.057

[67] Lamia KA , Sachdeva UM , DiTacchio L , Williams EC , Alvarez JG , Egan DF , Vasquez DS , Juguilon H , Panda S , Shaw RJ *et al*. (2009) AMPK regulates the circadian clock by cryptochrome phosphorylation and degradation. Science 326, 437–440. 19833968 10.1126/science.1172156 PMC2819106

[68] Angleys H & Ostergaard L (2022) Modeling the measurement bias in interstitial glucose concentrations derived from microdialysis in skeletal muscle. Physiol Rep 10, e15252. 35439357 10.14814/phy2.15252 PMC9017984

[69] Gudbjornsdottir S , Sjostrand M , Strindberg L , Wahren J & Lonnroth P (2003) Direct measurements of the permeability surface area for insulin and glucose in human skeletal muscle. J Clin Endocrinol Metab 88, 4559–4564. 14557422 10.1210/jc.2003-030434

[70] Maggs DG , Jacob R , Rife F , Lange R , Leone P , During MJ , Tamborlane WV & Sherwin RS (1995) Interstitial fluid concentrations of glycerol, glucose, and amino acids in human quadricep muscle and adipose tissue. Evidence for significant lipolysis in skeletal muscle. J Clin Invest 96, 370–377. doi: 10.1172/JCI118043 7615807 PMC185209

[71] Qian X , Esteban L , Vass WC , Upadhyaya C , Papageorge AG , Yienger K , Ward JM , Lowy DR & Santos E (2000) The Sos1 and Sos2 Ras‐specific exchange factors: differences in placental expression and signaling properties. EMBO J 19, 642–654. 10675333 10.1093/emboj/19.4.642 PMC305602

[72] Baltanas FC , Zarich N , Rojas‐Cabaneros JM & Santos E (2020) SOS GEFs in health and disease. Biochim Biophys Acta Rev Cancer 1874, 188445. doi: 10.1016/j.bbcan.2020.188445 33035641

[73] Garcia‐Navas R , Liceras‐Boillos P , Gomez C , Baltanas FC , Calzada N , Nuevo‐Tapioles C , Cuezva JM & Santos E (2021) Critical requirement of SOS1 RAS‐GEF function for mitochondrial dynamics, metabolism, and redox homeostasis. Oncogene 40, 4538–4551. 34120142 10.1038/s41388-021-01886-3 PMC8266680

[74] Peeters K , Van Leemputte F , Fischer B , Bonini BM , Quezada H , Tsytlonok M , Haesen D , Vanthienen W , Bernardes N , Gonzalez‐Blas CB *et al*. (2017) Fructose‐1,6‐bisphosphate couples glycolytic flux to activation of Ras. Nat Commun 8, 922. 29030545 10.1038/s41467-017-01019-z PMC5640605

[75] Corral‐Ramos C , Barrios R , Ayte J & Hidalgo E (2022) TOR and MAP kinase pathways synergistically regulate autophagy in response to nutrient depletion in fission yeast. Autophagy 18, 375–390. 34157946 10.1080/15548627.2021.1935522 PMC8942533

[76] Wei Y , An Z , Zou Z , Sumpter R , Su M , Zang X , Sinha S , Gaestel M & Levine B (2015) The stress‐responsive kinases MAPKAPK2/MAPKAPK3 activate starvation‐induced autophagy through Beclin 1 phosphorylation. eLife 4, e05289. doi: 10.7554/eLife.05289 25693418 PMC4337728

[77] Lauinger L , Diernfellner A , Falk S & Brunner M (2014) The RNA helicase FRH is an ATP‐dependent regulator of CK1a in the circadian clock of *Neurospora crassa* . Nat Commun 5, 3598. 24710172 10.1038/ncomms4598

[78] Sancar G , Sancar C , Brugger B , Ha N , Sachsenheimer T , Gin E , Wdowik S , Lohmann I , Wieland F , Hofer T *et al*. (2011) A global circadian repressor controls antiphasic expression of metabolic genes in Neurospora. Mol Cell 44, 687–697. 22152473 10.1016/j.molcel.2011.10.019

[79] McCluskey K (2003) The fungal genetics stock center: from molds to molecules. Adv Appl Microbiol 52, 245–262. 12964247 10.1016/s0065-2164(03)01010-4

[80] Margolin B , Freitag M & Selker E (1997) Improved plasmids for gene targeting at the his‐3 locus of *Neurospora crassa* by electroporation. Fungal Genet Biol 44, 34–36. doi: 10.4148/1941-4765.1281

[81] Vogel H (1964) Distribution of lysine pathways among fungi: evolutionary implications. Am Nat 98, 435–446. doi: 10.1086/282338

[82] Malzahn E , Ciprianidis S , Kaldi K , Schafmeier T & Brunner M (2010) Photoadaptation in Neurospora by competitive interaction of activating and inhibitory LOV domains. Cell 142, 762–772. 20813262 10.1016/j.cell.2010.08.010

[83] Luo CH , Loros JJ & Dunlap JC (1998) Nuclear localization is required for function of the essential clock protein FRQ. EMBO J 17, 1228–1235. doi: 10.1093/emboj/17.5.1228 9482720 PMC1170471

[84] Henriques AF , Jenkins RE , Burton NF & Cooper RA (2010) The intracellular effects of manuka honey on Staphylococcus aureus. Eur J Clin Microbiol Infect Dis 29, 45–50. 19813035 10.1007/s10096-009-0817-2

[85] Hubner NC , Bird AW , Cox J , Splettstoesser B , Bandilla P , Poser I , Hyman A & Mann M (2010) Quantitative proteomics combined with BAC TransgeneOmics reveals in vivo protein interactions. J Cell Biol 189, 739–754. 20479470 10.1083/jcb.200911091 PMC2872919

[86] Lang L , Pettko‐Szandtner A , Tuncay Elbasi H , Takatsuka H , Nomoto Y , Zaki A , Dorokhov S , De Jaeger G , Eeckhout D , Ito M *et al*. (2021) The DREAM complex represses growth in response to DNA damage in Arabidopsis. Life Sci Alliance 4, e202101141. 34583930 10.26508/lsa.202101141 PMC8500230

[87] Pettkó‐Szandtner A , Magyar Z & Komaki S (2024) Functional framework of the kinetochore and spindle assembly checkpoint in *Arabidopsis thaliana*. *bioRxiv* . 10.1093/plphys/kiaf461 PMC12526889 41022395

[88] Ughy B , Nagyapati S , Lajko DB , Letoha T , Prohaszka A , Deeb D , Der A , Pettko‐Szandtner A & Szilak L (2023) Reconsidering dogmas about the growth of bacterial populations. Cells 12, 1430. 37408264 10.3390/cells12101430 PMC10217356

[89] Robinson MD , McCarthy DJ & Smyth GK (2010) edgeR: a Bioconductor package for differential expression analysis of digital gene expression data. Bioinformatics 26, 139–140. 19910308 10.1093/bioinformatics/btp616 PMC2796818

[90] Branson OE & Freitas MA (2016) A multi‐model statistical approach for proteomic spectral count quantitation. J Proteomics 144, 23–32. 27260494 10.1016/j.jprot.2016.05.032 PMC4967010

[91] Perez‐Riverol Y , Bandla C , Kundu DJ , Kamatchinathan S , Bai J , Hewapathirana S , John NS , Prakash A , Walzer M , Wang S *et al*. (2025) The PRIDE database at 20 years: 2025 update. Nucleic Acids Res 53, D543–D553. 39494541 10.1093/nar/gkae1011 PMC11701690

