# Supplementary material for: Conserved function of a RasGEF‐mediated pathway in the metabolic compensation of the circadian clock
Source: FEBS J. 2025 May 2;292(20):5335–54. doi: 10.1111/febs.70122 (PMC12525002; doi:10.1111/febs.70122)
Supplement: Supplementary file 1 — Fig. S1. The growth and circadian phenotype of Δrasgef. Table S1. List of the proteins with RasGEFFlag‐specific enrichment according to the criteria detailed in the Materials and Methods section. [file FEBS-292-5335-s001.pdf]

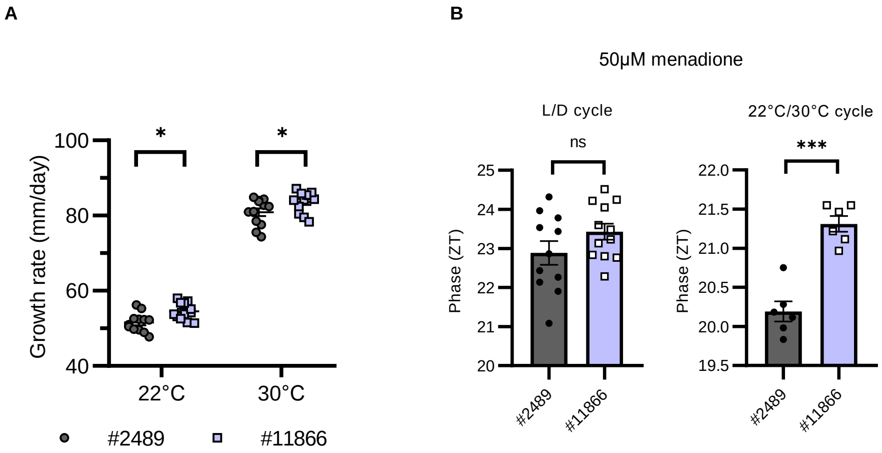

Fig S1. The growth and circadian phenotype of *Δrasgef*

(A) *Δrasgef* (#11866) grows faster than the control (#2489) strain. Race tubes were inoculated with the indicated conidia and incubated at 22°C and 30°C under 12/12 hour light/dark (L/D) cycles. Growth front was marked at specific time points and growth rate was calculated (n = 12). (B) Delayed phase of conidiation in *Δrasgef* (#11866) under temperature cycles. Race tubes containing 50 μM menadione were inoculated with the indicated conidia and incubated at 25°C under 12/12 hour L/D (left panel) or 22°C/30°C temperature cycles (right panel), as indicated. Circadian phase of conidiation peaks was calculated with the Chrono program (n = 11 (#2489) and 12 (#11866) in the L/D cycle, n = 6 in the 22°C/30°C cycle).

All data were presented as mean ± SEM. Statistical analysis was performed using two-way ANOVA followed by Fisher's LSD test (A) and two-sample t-test (B). ns: not significant; *$p < 0.05$; ***$p < 0.001$.

Table S1. List of the proteins with RasGEF$_{Flag}$-specific enrichment according to the criteria detailed in the Materials and methods section

Protein ID	Protein Name	logFC	p value	FDR
V5IQC8	Ras guanine-nucleotide exchange protein	16,2603248	5,02E-39	2,06E-35
Q7S5W4	Centrin 3	11,3046464	1,43E-05	9,67E-04
U9W4J8	DUF1941-domain-containing protein	10,5630865	1,66E-11	3,40E-08
A0A0B0DTI2	Acyltransferase-domain-containing protein	9,98064611	9,01E-10	1,23E-06
Q7S8G8	Pyridoxamine phosphate oxidase	9,72527236	1,09E-07	3,42E-05
U9W2W2	DUF1421 multi-domain protein	9,6381016	3,75E-05	1,99E-03
P23622	Sulfate permease 2	9,58104403	1,40E-09	1,43E-06
Q7S5H7	GTPase Ras2p	9,50075136	1,09E-06	1,55E-04
Q7RXZ8	Acetoacetyl-CoA synthase	9,32977945	5,92E-09	4,04E-06
V5IMI3	Heme peroxidase	9,19292311	7,70E-07	1,17E-04
A0A0B0DKW5	Heme peroxidase	9,19292311	7,70E-07	1,17E-04
Q7S2C8	Late endosomal/lysosomal adaptor and MAPK and MTOR activator 1	9,15295011	0,00336377	0,04767468
Q7SE88	RRM domain-containing protein	9,12926113	2,29E-05	1,30E-03
Q1K7M0	Vacuolar membrane protease	9,07431149	6,45E-07	1,06E-04
Q6MVU1	Uncharacterized protein B15B3.230	9,07036408	2,99E-08	1,36E-05
Q6MVK4	Related to calcium-independent phospholipase A2	9,04868792	0,00056054	0,01481272
Q6MUS6	Probable kinesin-related protein bimC	8,93006801	6,61E-05	3,01E-03
Q7RY33	Carrier domain-containing protein	8,91107689	3,51E-07	7,19E-05
Q6MGE5	Related to amidase	8,90785564	0,00021193	7,29E-03
Q7RXY8	Membrane-associated, eicosanoid/glutathione metabolism (MAPEG) protein	8,82058726	8,24E-06	6,90E-04
Q7SEK3	Hyphal anastamosis-8 protein	8,80954264	2,40E-07	5,78E-05
A0A0B0DSQ1	Carrier domain-containing protein	8,80834091	4,97E-07	9,26E-05
Q7SE06	Altered inheritance of mitochondria protein 11	8,70009885	0,00586534	0,06702742
Q1K588	Calcium-transporting ATPase	8,68950269	5,08E-06	5,10E-04
Q7S8T9	DNA repair and recombination protein RAD5C	8,63079654	3,68E-07	7,19E-05
Q7S289	NmrA family protein	8,59733146	0,00028135	0,00893342
V5IN16	Peroxisomal biogenesis factor	8,55687452	1,54E-06	1,97E-04
Q8NIX5	Uncharacterized protein 62D11.090	8,54935062	0,00618123	0,0687998
Q7S6D1	Modin	8,53214715	6,21E-08	2,31E-05
U9W2Y6	Yippee zinc-binding protein	8,50380739	6,06E-05	0,00288726
Q7SGU4	Spindle pole body protein ppc89	8,50216365	5,43E-06	0,00052924
Q7SD18	Peroxisomal membrane protein PEX16	8,44018026	1,16E-06	1,58E-04
V5IP78	Multidrug resistance protein 3, variant 1	8,4371698	1,52E-05	9,67E-04
Q7SA49	Chromosome transmission fidelity protein 18	8,43658369	0,00087671	0,01995006
Q7S3P3	FAD-binding FR-type domain-containing protein	8,42420452	1,44E-05	9,67E-04
V5IPV8	Complex1_LYR_dom domain-containing protein	8,40897285	0,00243448	0,03917327
Q1K834	T5orf172 domain-containing protein	8,40511332	0,00124063	0,02566485
Q6MV56	Uncharacterized protein 53H1.020	8,35741407	0,00012715	0,00500764
Q1K6J8	Phosphatidic acid phosphatase beta	8,32050528	8,26E-06	6,90E-04
Q7RZE5	C3HC-type domain-containing protein	8,28826717	0,0024525	0,03917327
Q872W6	Uncharacterized protein B2G14.020	8,26901392	0,00705987	0,07491508
Q7RVS1	Multidrug resistance protein MDR	8,25784216	1,71E-05	1,03E-03
Q7SFN5	pH-response regulator protein palI/prr-5	8,1846646	8,92E-06	7,17E-04
O93835	Fr protein	8,17470917	8,29E-05	3,50E-03
Q7RYC0	Cobalamin biosynthesis protein CbiB	8,15624896	0,00010803	0,00438113
Q7SDX8	RING-1	8,13368733	0,00655694	0,07170557

Q7S1P9	DNA repair protein rad5	8,10796873	0,00262793	0,04124136
Q7SAC5	Tartrate transporter	8,10485268	3,68E-07	7,19E-05
Q7RZP8	Peroxisomal adenine nucleotide transporter 1	8,10201209	0,00675597	0,0726311
Q7S4B9	Clr5 domain-containing protein	8,09027193	8,90E-07	0,00013014
Q9P6D7	Acyl-CoA binding protein	8,02534006	0,00119455	0,02522111
Q1K720	Spindle pole body component	8,01298257	5,11E-06	5,10E-04
Q1K4T6	CMP/dCMP-type deaminase domain-containing protein	8,0123529	0,00013834	5,31E-03
Q7S285	Patatin-like phospholipase domain-containing protein	7,94837988	3,81E-05	0,00200144
Q7S9T1	Metallophosphoesterase domain-containing protein 2	7,94561935	6,84E-05	3,05E-03
Q7SCJ2	AB hydrolase-1 domain-containing protein	7,93406082	1,35E-05	9,44E-04
Q7S6W0	Uncharacterized protein	7,9099381	0,00758665	0,07807764
Q1K7W2	Cohesin loading factor-domain-containing protein	7,89522403	0,00287227	0,04373538
Q9UV10	Heterokaryon incompatibility protein 6, OR allele	7,86437026	0,00017249	0,00636515
A0A0B0EDF5	Non-specific serine/threonine protein kinase	7,86115954	6,59E-06	6,07E-04
A7UVV8	Non-specific serine/threonine protein kinase	7,86115954	6,59E-06	6,07E-04
Q7RYR0	ANK_REP_REGION domain-containing protein	7,86019633	2,03E-06	2,52E-04
U9W8Y7	PIPK domain-containing protein	7,80425417	0,00017463	0,00638645
Q6M9F1	SUN domain-containing protein	7,79532044	0,00020866	0,00724299
Q7S8U0	Peroxin-12	7,76663192	0,00465626	0,05811095
Q7S9J5	Vacuolar calcium ion transporter/H(+) exchanger	7,72316558	1,31E-06	1,73E-04
Q35141	NADH-ubiquinone oxidoreductase chain 3	7,68649967	1,16E-05	0,00086235
Q7RW81	GTPase activating protein	7,68608836	2,90E-08	1,36E-05
Q6MV54	Peroxin-13	7,65317208	0,00442709	0,05596374
Q7RUV9	MPN domain-containing protein	7,64424794	0,00931782	0,08594301
Q7SEL9	Triacylglycerol lipase	7,6266143	0,00021987	0,00737305
Q7SHA5	Lipase_3 domain-containing protein	7,62242425	7,28E-06	0,00064788
Q1K4V5	Cauli_VI domain-containing protein	7,6095739	0,00907065	0,08482505
Q872N4	Uncharacterized protein B19A17.180	7,58686476	3,01E-06	3,33E-04
V5IRK5	Zn(2)-C6 fungal-type domain-containing protein	7,55416537	5,35E-09	4,04E-06
Q6MV76	Uncharacterized protein B5K2.080	7,54765377	1,49E-05	0,00096668
Q1XH09	Non-specific serine/threonine protein kinase	7,54560116	1,18E-05	8,64E-04
Q7SDL7	Mitochondrial genome maintenance protein Mgr2	7,53394215	0,00918315	0,08554972
Q9P3H4	HET-domain-containing protein	7,51565636	0,00024629	0,00800632
Q6MGH8	Related to NAD(P)H-dependent oxidoreductase	7,50847055	0,0006473	0,01665586
Q01277	Probable E3 ubiquitin ligase complex SCF subunit scon-2	7,47278259	0,00368227	0,04977746
Q6MUU5	Related to TFIID and SAGA subunit	7,46655681	2,87E-06	3,27E-04
Q7S6P0	TORC1 subunit TCO89	7,46180652	0,01265012	0,10092799
Q1K6T6	J domain-containing protein	7,43338626	0,00375295	0,05007199
Q6MV18	Uncharacterized protein B16B8.230	7,43068875	0,00010743	0,00438113
Q1K7Q9	P-loop containing nucleoside triphosphate hydrolase protein	7,42591528	0,01045238	0,09148069
V5ING0	C6 transcription factor	7,40167834	7,60E-05	3,28E-03
Q6MVG3	Signal peptidase complex subunit 2	7,3900159	0,00700722	0,07474373
Q7S902	Spindle pole body component	7,38191758	0,0003217	0,00990733
Q7SGR4	DUF1691 domain-containing protein	7,38046989	0,00405342	0,05293023
V5INC4	WD repeat protein	7,37960511	2,50E-05	1,37E-03
Q7S9F2	YL1_C domain-containing protein	7,37854167	0,01245121	0,10059204
Q7SDL6	FHA domain-containing protein	7,36941522	0,01078045	0,09315761
Q7S2X6	Moz represents a chromatin-associated acetyltransferase	7,35568858	0,01087443	0,09357492

Q1K722	Pheromone-processing carboxypeptidase kex1	7,33763519	0,00137211	0,02782258
Q7S190	HET domain-containing protein	7,33055044	8,25E-08	2,82E-05
Q7RYT2	Probable lysosomal cobalamin transporter	7,32856882	0,01043742	0,09148069
Q7SA25	Alpha-1,6-mannosyltransferase Och1	7,31381274	0,00044288	0,01242488
Q7SF08	Proteasome activator subunit 3/P28 gamma	7,29090654	3,45E-06	0,00037223
Q7SEA6	Peroxisomal membrane protein	7,28720935	0,01268008	0,10092799
Q1K7R2	Nuclear migration protein	7,28271628	7,58E-06	6,60E-04
A0A0B0DQX0	RRM domain-containing protein	7,27789424	0,00071972	0,01754744
Q7S2M1	SWI5-dependent HO expression protein 3	7,25839493	0,00326506	0,04692528
Q7RW66	DEAD box family helicase	7,22512885	0,00518465	0,06155454
Q873I1	HET-domain-containing protein	7,21741474	0,00035385	0,01057939
Q7S6B3	BEAK-2	7,20886489	0,00167813	0,03167572
Q7S9N8	Autophagy-related protein	7,19595403	0,00159958	0,03090502
Q7S150	Glyco_trans_2-like domain-containing protein	7,18928638	0,0044275	0,05596374
Q7S4W4	Uncharacterized protein	7,18460007	0,00112775	0,02444048
Q1K8Z3	Syntaxin	7,18177237	0,01221031	0,0992629
V5IQH2	C2 NT-type domain-containing protein	7,17319822	0,00188937	0,03364716
Q7S4E1	DUF2013 domain-containing protein	7,15095148	0,0015371	0,02998077
Q7S5X2	Methyltransferase-UbiE family protein	7,15017581	0,01303882	0,10211661
Q6MVF5	Related to glutaredoxin	7,14741315	0,01180207	0,09759103
Q7S864	TFCD_C domain-containing protein	7,13921125	0,01159983	0,09696511
Q7S718	Leucine-rich repeat-containing protein 40	7,11461734	0,00013869	5,31E-03
Q1K7K2	Csi2 protein	7,08106697	0,00719254	0,07515471
Q7RYD3	Sulfite oxidase	7,07463817	5,85E-08	2,31E-05
Q6MFE7	DUF155 domain-containing protein	7,0701731	0,01252033	0,10075298
Q6MV27	Uncharacterized protein B16B8.140	7,06750035	0,00045397	0,0125638
Q6MFD8	Kinetochore protein Mis12/MTW1	7,026352	0,00494731	0,06020203
Q1K5F8	CRC domain-containing protein	7,00988807	0,01205687	0,0985727
Q7RXL3	DUF788 domain protein	7,00436377	0,01249115	0,10071606
Q1K776	Transcription factor	6,98175104	1,69E-05	0,00103007
Q6MFM6	Structure-specific endonuclease subunit SLX4	6,97798271	0,01640506	0,11410825
Q872V2	Uncharacterized protein B23G1.020	6,95368287	0,00233958	0,03864079
Q7SBC8	DNA repair protein Rhp26/Rad26	6,93681238	0,00069728	0,01730942
Q7S8K3	Spindle pole body component	6,91277573	0,01292648	0,10200934
Q7S3L8	Nickel/cobalt efflux system	6,90403625	0,01424895	0,10650313
Q1K5D6	DUF3669 domain-containing protein	6,89875484	0,01575111	0,11200786
Q7SEM7	Alpha-1,3-glucosyltransferase	6,88514672	0,00217353	0,0372501
Q7S3T6	Integral membrane protein	6,87950972	7,58E-05	3,28E-03
Q1K585	Cation diffusion facilitator 10	6,85733572	0,00133733	0,02725216
Q01285	Chitin synthase 4	6,85635228	0,00058118	0,01516248
Q7SAS3	Component of oligomeric Golgi complex 7	6,85467241	0,01682388	0,11591125
Q6MGG9	Enolase	6,84009172	0,0155078	0,11135948
Q7S126	DNA polymerase epsilon subunit B	6,83969371	0,01471271	0,10885068
Q9HEF8	Rhomboid-like protein	6,83515662	0,01327218	0,10290081
Q7S5D5	Monooxygenase	6,82054107	0,00044797	1,25E-02
Q7S0R1	Cyclin-domain-containing protein	6,82035931	0,00138297	0,02790464
V5IQB8	HET domain-containing protein	6,78758643	0,00832852	0,08200854
Q7SHI9	Rho-GAP domain-containing protein	6,78022207	0,00273075	0,04252917
Q7SGU0	Mitochondrial ribosomal protein subunit S18	6,7470059	0,00161296	0,03100254
Q1K6D7	Brr6_like_C_C domain-containing protein	6,73839422	0,00031422	9,75E-03

Q1K4W4	ARS-binding protein 2	6,73797442	0,01553347	0,11135948
Q1K6B1	Pentatricopeptide repeat protein	6,73622113	0,00319729	0,04677178
Q7SF24	Copper transport protein	6,68167495	0,01434863	0,10666419
Q6MFH0	Lipase_3 domain-containing protein	6,67875996	1,82E-07	5,34E-05
Q7RVU8	Ubiquinone biosynthesis protein	6,67158151	0,01563984	0,11179889
Q9P388	Related to small s protein	6,65798362	5,41E-05	2,67E-03
Q7SAP3	RING-type domain-containing protein	6,64995988	0,02264395	0,13376982
Q7S7J6	Signal peptidase subunit 3	6,64737762	0,01437917	0,10669759
Q1K5E5	Serine/threonine protein kinase	6,63985544	0,00111392	2,44E-02
Q7S1Q4	ATPase expression protein 2, mitochondrial	6,60884919	0,00174821	0,0319201
Q1K606	HECT domain-containing protein	6,58970209	0,00079196	1,86E-02
Q7SI21	RING-13 protein	6,5873401	6,23E-07	1,06E-04
A0A0B0EGD6	ATP-dependent DNA helicase	6,5873401	6,23E-07	0,00010567
Q6MFU0	Probable succinate-fumarate transporter	6,57538149	0,01738241	0,1171037
Q1K6C2	Repressor of RNA polymerase III transcription MAF1	6,55861538	0,00119069	0,02522111
Q7SD73	Rif1_N domain-containing protein	6,5440253	0,00736264	0,07673636
V5IPM7	Transmembrane protein	6,54092707	0,01673865	0,11591125
Q7RYK8	CLC channel protein	6,53590338	0,00057351	1,51E-02
V5IN74	Protein kinase, variant	6,52176335	0,00112757	0,02444048
Q7RY31	DUF1881 domain-containing protein	6,51227617	0,00656484	0,07170557
Q7SC58	PH domain-containing protein	6,50042715	0,01621122	0,11389566
Q7SDI4	Kinesin	6,47363744	2,55E-06	2,99E-04
Q7S6M2	WAPL domain-containing protein	6,46653016	0,01109023	0,09469391
Q7S2G0	C6 finger domain-containing protein	6,45459688	0,00030434	0,00951592
Q7SE08	Phosphoinositide phospholipase C	6,45191158	0,01919146	0,12314115
V5IQY8	DNA repair exonuclease rad1	6,44003021	0,00325075	0,04688406
Q6MW34	Uncharacterized protein B13M13.090	6,43171722	0,01748631	0,1171037
V5IKT6	WSC-1	6,42224746	0,01725442	0,1171037
Q7S4P5	DUF1917-domain-containing protein	6,40454184	0,00830303	0,08200854
A0A0B0DMF4	DUF1917-domain-containing protein	6,40454184	0,00830303	0,08200854
Q7RWW1	CENP-V/GFA domain-containing protein	6,40399548	0,01689429	0,11591125
Q1K622	Female fertility-7	6,3996286	0,0171335	0,1171037
V5IQI1	Beta-lactamase-like protein	6,37691805	0,01686604	0,11591125
V5ILN0	Zn(2)-C6 fungal-type domain-containing protein	6,36946507	0,00374829	0,05007199
Q1K8I6	NACHT domain-containing protein	6,36186286	0,00279242	0,04298356
Q7SC91	Probable tRNA-splicing endonuclease subunit tsp-5	6,35346983	0,01746709	0,1171037
Q1K7Y4	RING finger protein	6,32908865	0,00196478	0,03453957
Q1K6D5	MFS general substrate transporter	6,32795641	2,42E-05	1,34E-03
Q7S5M7	Cytochrome c oxidase subunit 9, mitochondrial	6,32298007	0,00663596	0,07208237
Q7S7B7	Oxidoreductase	6,31029657	0,01851437	0,12075612
A0A0B0DSU8	2-nitropropane dioxygenase	6,31029657	0,01851437	0,12075612
V5IP23	Interferon-induced 6-16	6,31013261	0,0190356	0,12278708
Q7S1A9	AAA domain-containing protein	6,30188676	3,63E-07	7,19E-05
Q7SB66	Peroxin 11c	6,30126615	0,00021687	0,00734126
Q7RZT9	Serine/threonine-protein kinase tel1	6,29338884	0,01746682	0,1171037
Q7S0I5	MFS monosaccharide transporter	6,28764334	0,01947742	0,12425986
V5IR72	CCCH zinc finger protein, variant	6,28517122	0,01822058	0,11979373
Q7SCP3	Mannosyltransferase	6,28055646	0,01723734	0,1171037
Q6MW33	Uncharacterized protein B13M13.100	6,26937861	0,01715735	0,1171037
V5ILK3	Protein transport protein sec73	6,26195583	1,79E-05	0,00106468

Q7RY73	Peroxin 8	6,25013345	0,00025372	0,00818296
Q870V1	Uncharacterized protein B14A21.210	6,24623336	0,0058463	0,06702742
Q7S8M7	Mitochondrial fission process protein 1	6,23653826	0,01749694	0,1171037
V5IPA3	Integral membrane protein	6,21355029	0,01986605	0,12461155
Q7S6C3	Amino-acid permease inda1	6,16068534	0,01254897	0,10078547
U9W3A4	RING-type domain-containing protein	6,15809204	0,01971671	0,12436488
Q7S9G1	Dolichyl-phosphate beta-glucosyltransferase	6,15466395	0,00929058	0,08594301
Q7S6M4	PROTEASOME_ALPHA_1 domain-containing protein	6,11918294	0,02242598	0,13312584
Q7SCX0	Vacuolar transporter chaperone 4	6,10936181	0,00193074	0,03408757
Q871W2	Related to acetyl coenzyme A synthetase	6,08808863	0,03995024	0,17690399
Q1K666	NIMA interactive protein	6,08743448	0,00429052	0,05509087
Q1K539	Mitochondrial carrier	6,08339936	0,00014178	0,00533381
Q7S893	Sister chromatid cohesion protein	6,0810562	9,40E-06	0,00074047
Q7SF27	P-loop containing nucleoside triphosphate hydrolase protein	6,07328737	4,74E-06	4,98E-04
Q7RYD4	Polysaccharide export protein	6,06923106	0,00832899	0,08200854
Q7SGN9	Restriction of telomere capping protein 4	6,06280125	0,02122623	0,12884425
Q7S6X2	ABC transporter CDR4	6,04830788	0,00631231	0,06978564
Q7RY51	Histone transcription regulator 3 homolog	6,04604255	0,00174468	0,0319201
Q1K578	General transcription and DNA repair factor IIH	6,04063726	0,00523629	0,06198793
Q1K533	Uncharacterized protein	6,03858523	0,02092182	0,12790417
Q1K4U1	Transcription factor TFIID complex subunit Taf13	6,03698307	0,02001163	0,12533275
Q7S473	Microtubule associated protein	6,03593826	1,35E-05	0,00094418
Q1K6Q2	BZIP domain-containing protein	6,03529577	0,01877309	0,121531
V5IR03	Kinesin	6,01916941	2,05E-07	5,51E-05
Q6M9F4	ANK_REP_REGION domain-containing protein	6,01712556	0,00161976	0,03100254
Q6MVB3	Related to cullulin 3	6,0165375	6,29E-05	2,94E-03
Q7S102	Lathosterol oxidase	6,00184942	0,01902561	0,12278708
Q7S3Y7	Protein SQS1	5,97324157	0,00246476	0,03917327
Q7S9J1	Glycosyltransferase 2	5,97080491	1,01E-05	0,00076949
Q7SDB1	Conserved glycine-rich protein	5,94513436	0,01028861	0,09109381
V5IQM7	Spa2-like protein	5,94250916	0,00100075	0,02227753
P0C584	Patatin-like phospholipase domain-containing protein NCU11180	5,92619816	0,00026372	0,00843908
Q6MV34	Uncharacterized protein B16B8.060	5,91206574	0,00232088	0,03848713
O74693	Clock-controlled gene-9 protein	5,90503099	0,02430514	0,1365622
Q1K656	Kinesin-like protein	5,87679773	0,02065046	0,1271944
Q7SHU4	VPS13_C domain-containing protein	5,87240755	0,00989739	0,08890283
Q6M950	CENP-C_C domain-containing protein	5,86797425	0,00363899	0,04953436
Q7S0E3	Glucan 1,3-beta-glucosidase	5,8635599	0,02348239	0,13483287
Q6M913	Transmembrane protein	5,86191813	0,02158461	0,12982461
P48479	G2-specific protein kinase nim-1	5,86034083	0,01203203	0,09856642
Q7SBI0	Vacuolar protein sorting-associated protein 45	5,85273593	2,39E-05	0,0013397
Q1K553	Division septum protein Blr	5,85052872	0,02014301	0,12577102
Q7RXR3	Histone H3-like centromeric protein hH3v	5,83038405	0,02277305	0,1338284
V5IKK6	AAA family ATPase	5,8257893	0,00534621	0,06310686
Q7SDN3	Probable lysosomal cobalamin transporter	5,82470621	0,00077647	0,0184634
Q6MUS2	Uncharacterized protein G17B7.090	5,81812942	0,02050708	0,1265015
Q7SGS0	RGS domain-containing protein	5,80854314	0,00801121	0,0811248
Q873A9	Uncharacterized protein B24N11.150	5,80244339	0,02310421	0,13389264
Q1K6Y8	LIM domain protein	5,7978921	0,00172081	0,0319201

Q1K7P2	RNI-like protein	5,78725416	0,00864314	0,08310399
Q7S114	Peroxisomal adenine nucleotide transporter 1	5,7867596	0,01329107	0,10290081
Q7RXR2	Derlin	5,7773672	0,00872718	0,08351996
Q7SFN4	ABC transporter	5,75968607	0,03871183	0,17447688
Q7S9A4	ANK_REP_REGION domain-containing protein	5,75813561	0,02184301	0,13061163
Q9P3N8	Uncharacterized protein B7F18.110	5,7519601	0,02086353	0,12790417
P38670	Delta(14)-sterol reductase	5,74703932	2,60E-05	0,00140357
Q7SHR6	DUF1713 domain-containing protein	5,73840542	0,0048872	0,05975517
V5IMP0	YTH domain-containing protein	5,71961192	0,024234	0,13651156
Q9C0R0	TIM23	5,71867803	0,02351608	0,13483287
V5INW2	Rho GTPase activator	5,71650388	0,00225694	0,03806995
Q7S0P5	G domain-containing protein	5,69992606	1,36E-05	9,44E-04
Q7RV33	Hormone-sensitive lipase	5,69880431	0,00068456	1,71E-02
Q7RWS7	PHD finger domain-containing protein	5,68071007	0,01188901	0,09759103
Q9P353	Maintenance of mitochondrial morphology protein 1	5,67829907	0,02168081	0,13021204
Q1K8K7	Short chain dehydrogenase/reductase	5,667743	0,02776631	0,14469569
Q7S5R0	Oligosaccharide translocation protein RFT1	5,66732397	0,02183377	0,13061163
Q7S8N5	BUD3	5,66335089	0,00116934	2,50E-02
Q6MV60	Uncharacterized protein B5K2.240	5,66278354	0,01718505	0,1171037
Q7S2Z7	TPR_REGION domain-containing protein	5,6582323	0,00175342	0,0319201
P0CY45	NADH-ubiquinone oxidoreductase chain 6	5,65804066	0,00554155	0,06503783
Q7S041	SAM and PH domain-containing protein	5,63159866	0,02280735	0,13383797
Q6MFU1	Related to hard surface induced protein 3	5,62839385	0,00250614	0,03963371
Q9P6D6	Related to Ribonuclease III	5,62591849	0,02466954	0,13747812
Q7RY40	Transcription factor IIIB 90 kDa subunit	5,62513285	0,00048263	1,31E-02
U9W4K5	DSBA family oxidoreductase	5,61730574	0,01400227	0,10523541
Q6MWQ1	Uncharacterized protein B24N4.160	5,616183	0,02560998	0,13980585
Q1K4S8	Related to transport protein USO1	5,60959604	0,00035018	0,01054674
Q6MUM9	DNA-directed RNA polymerase subunit	5,58588841	0,01014306	0,09031734
Q7SAC4	Chromatin remodeling factor 5-1	5,57715454	0,00022901	0,00755499
Q870U5	Uncharacterized protein B11H7.010	5,57627714	0,02295618	0,13389264
Q871M6	Cation-transporting ATPase	5,56992038	0,00635989	0,07002722
Q872S3	Protein OS-9 homolog	5,56484955	0,01226012	0,0992629
Q7S882	Oligopeptide transporter 2	5,54907549	0,00013359	0,00521113
Q1K7W1	Rhomboid-like protein	5,54669076	0,02593326	0,14042378
A0A0B0DYV6	Rhomboid-like protein	5,54669076	0,02593326	0,14042378
Q1K921	Aromatic ring-opening dioxygenase LigB subunit	5,54501929	0,02493524	0,13781798
Q6MV69	Uncharacterized protein B5K2.150	5,54309363	0,04205611	0,18189704
Q7SFR3	Non-specific serine/threonine protein kinase	5,5367709	0,02563335	0,13980585
Q870V7	Protein PNS1	5,53667188	0,01620515	0,11389566
Q1K6R3	C2H2-type domain-containing protein	5,53142144	0,01188063	0,09759103
Q7SHT9	Rho GAP domain-containing protein	5,52758517	8,41E-05	0,00351481
Q1K589	Carbohydrate O-acetyltransferase	5,51617073	0,01223775	0,0992629
V5IQL0	Urea transporter	5,47739221	0,0129504	0,10200934
Q7S513	Calcofluor white hypersensitive protein	5,46525881	0,0242628	0,13651156
Q1K8V6	HET domain-containing protein	5,43523195	0,02442419	0,13690512
Q7SFE2	RAB family GTPase	5,42356016	0,01539479	0,11101594
U9W842	CaaX prenyl proteinase Rce1	5,41851928	0,02949072	0,1491284
A0A0B0DVM5	Kinase-like protein	5,4165964	0,00306631	0,046019
Q7SAH4	Mutator-like element	5,41344827	0,01262857	0,10092799

V5IKP7	Amino acid permease	5,40038131	0,00179866	0,0325529
Q7S4N4	Regulatory factor Sgt1	5,3974471	0,0264781	0,14158524
Q1K758	Protein kinase domain-containing protein	5,39447712	0,01475861	0,10885068
Q8X0V8	Uncharacterized protein 123A4.340	5,39447712	0,01475861	0,10885068
Q7RXM1	Separase	5,38335876	0,02444522	0,13690512
Q7SGL9	Meiotically up-regulated 190 protein	5,37989518	0,0032361	0,0468674
U9W346	Xaa-Pro aminopeptidase	5,37760603	0,03931532	0,17595519
Q1K4R4	WAPL domain-containing protein	5,37232274	2,11E-05	0,00123414
Q7S2R0	MYND-type domain-containing protein	5,35198401	0,00230315	0,03841301
Q7S0S8	Cytochrome P450 3A4	5,34205681	0,00310042	0,04605633
Q7S7U0	Protein MAK16	5,33788128	0,02937953	0,14893384
Q7S020	Ubiquitin carboxyl-terminal hydrolase	5,29000606	0,01597588	0,11321317
Q7SD69	Peroxisomal half ABC transporter	5,2855715	6,66E-06	0,00060657
A0A0B0E939	UAA transporter	5,28524276	0,00313631	0,04620987
Q1K8H2	Mechanosensitive ion channel protein	5,25946222	0,00307842	0,046019
Q7S064	ANK_REP_REGION domain-containing protein	5,25852689	0,00052864	0,01415228
Q9P384	Uncharacterized protein B24M22.240	5,23865098	0,03787068	0,17293009
Q7S5F6	Siderophore iron transporter	5,22093094	0,02567448	0,13984396
Q7SER8	Triacylglycerol lipase	5,1981535	0,00063623	1,65E-02
Q6MGE0	Uncharacterized protein B13C5.150	5,18837104	0,02774732	0,14469569
Q7S3V4	ATP phosphoribosyltransferase	5,18699291	0,02625463	0,14113349
Q7SBI2	ATRX	5,18142152	0,0277256	0,14469569
Q7SHZ5	Non-structural maintenance of chromosomes element 4	5,1659446	0,03127605	0,15305462
Q8X001	Uncharacterized protein B23H20.120	5,15899739	0,01001197	0,0895394
Q1K734	Uncharacterized protein	5,15094544	0,03028358	0,15108594
Q7S5G8	Integral membrane protein	5,13610065	0,02873649	0,14689848
Q7SBW5	Vacuolar-sorting protein SNF8	5,13083078	0,00220483	0,03762902
Q1K4V6	BTB domain-containing protein	5,12953111	0,02978512	0,15004753
Q6MVM6	Uncharacterized protein B18P24.130	5,10359852	5,51E-05	2,69E-03
Q6IFT0	Putative two-component histidine kinase	5,089051	0,02867026	0,14689848
Q7S7G9	Chromodomain-helicase-DNA-binding protein 4	5,0817895	0,00020029	0,00707223
A0A0B0DGQ7	Chromatin remodeling factor mit1	5,0817895	0,00020029	0,00707223
Q7SEL7	Histone deacetylase	5,06425589	0,00075459	0,01807478
Q6MWS9	Uncharacterized protein B18P7.100	5,05617421	0,01772832	0,11826581
Q6MV09	Related to origin recognition complex subunit 3	5,05411414	0,01974003	0,12436488
Q7SEA2	Glycosyl transferase	5,047384	0,01337454	0,10297393
Q1K4T1	Sfi1 domain-containing protein	5,0470471	0,02922824	0,14853459
Q7SHT2	Telomere_reg-2 domain-containing protein	5,04599671	1,47E-05	0,00096668
Q7S1C6	Protein kinase domain-containing protein	5,04417401	0,00627199	0,06962074
Q6MGJ9	Related to transmembrane protein Tex-261	5,03926311	0,02800821	0,14542522
Q1K7W7	Transmembrane protein	5,03246918	0,0003291	0,01005975
Q7SI31	TORC1 growth control complex subunit Kog1	5,02065221	0,00369955	0,04978033
Q6MVZ1	Uncharacterized protein B11E5.250	5,00347552	0,03077614	0,15224527
Q1K563	AT hook domain-containing protein	4,99995437	0,0009593	0,0214715
Q1K4X1	Mitochondrial carrier	4,99412124	0,01714468	0,1171037
V5IMV4	NARG2_C domain-containing protein	4,99027956	0,00039572	0,01141464
Q7RWE4	Septin-type G domain-containing protein	4,95814891	0,00825588	0,08200854
Q7RX92	MFS transporter	4,9162966	0,00546899	0,06437059
Q7S0Y5	HET domain-containing protein	4,91115787	0,03322599	0,15954708

Q7SH39	Mitochondrial integral membrane protein	4,87513159	0,00036754	0,01083054
V5ILC2	DUF1691 domain-containing protein	4,87465928	0,03019105	0,1509921
Q7RYA2	Uncharacterized protein	4,86938663	0,01526698	0,11060314
A7UW65	MFS transporter Fmp42	4,86483258	0,03794363	0,1730703
Q7RZN2	DUF1736 domain-containing protein	4,85437321	0,03515566	0,16532442
Q7SA08	Hydrolase	4,85197859	0,0429198	0,1835068
V5IMD8	Ribosome biogenesis protein Urb1	4,85077722	0,00046048	0,01265855
Q7S8H7	ER membrane protein complex subunit 4	4,84996108	0,00871234	0,08351996
Q7SAX0	ANK_REP_REGION domain-containing protein	4,84072947	0,00709104	0,07505138
Q7S828	Lipase/serine esterase	4,8369667	0,01977157	0,12436488
Q7RXE4	Xanthine dehydrogenase	4,81033427	0,03965024	0,1764705
Q7S2N1	Stage V sporulation protein K	4,80988685	0,00017947	0,00650524
Q872C1	Uncharacterized protein X4G11.090	4,80165914	0,04785331	0,19495303
Q7SFX3	AAA domain-containing protein	4,79870505	0,03878615	0,17458032
Q7S9E4	Nuclear control of ATPase protein 2	4,7941131	0,01523994	0,11060314
Q6MVT7	Related to intracellular protein transport protein	4,79243749	0,00158355	0,03074036
V5IQW0	ING domain-containing protein	4,78714439	0,03730303	0,17148507
Q7SAS6	Integral membrane protein	4,78634206	0,03550442	0,16658204
P05510	NADH-ubiquinone oxidoreductase chain 5	4,77489197	0,03787056	0,17293009
V5IKZ9	PH domain-containing protein	4,77256357	0,0115095	0,09640681
Q7SA06	Inclusion body clearance protein iml2	4,76539848	0,00047779	1,30E-02
Q1K5T8	Cauli_VI domain-containing protein	4,73859894	0,01608108	0,11370424
Q7RWL8	Smr domain-containing protein	4,73828553	0,0096069	0,08711394
Q7S461	Rab-GAP TBC domain-containing protein	4,73400423	0,03347992	0,16039033
Q6M9A0	Zn(2)-C6 fungal-type domain-containing protein	4,72930996	0,03464135	0,16346884
Q7RX99	Serine/threonine-protein kinase apg-1	4,72735501	2,50E-06	2,99E-04
Q6MW11	Related to helicase-DNA-binding protein	4,71377043	0,0014128	0,02836672
Q7S7H8	WD domain-containing protein	4,71069957	0,03537244	0,16615311
Q7RXB6	Calmodulin	4,70253655	0,000189	6,79E-03
Q7S8P3	Ribonuclease P protein subunit	4,69010943	0,01741472	0,1171037
V5IRH4	Fungal_trans domain-containing protein	4,67581187	9,48E-05	0,00392311
Q7SE24	Protein prune homolog 2	4,67101531	0,03646575	0,16990643
Q6M900	Related to intermediate filament protein MDM1	4,66088505	0,00505421	0,06088837
Q7S478	Nuclear protein	4,65479935	0,01586538	0,11262492
V5IL70	Trafficking protein particle complex subunit 3, variant	4,63061442	0,00254571	4,01E-02
Q1K844	FAD/NAD(P)-binding domain-containing protein	4,61910413	0,00051619	0,01390987
Q6MVA9	Uncharacterized protein B1O14.060	4,58300446	0,00085059	0,01957318
Q7S856	DUF2415 domain-containing protein	4,56762988	0,0040405	0,05293023
Q1K611	C2H2-type domain-containing protein	4,56690875	0,02036863	0,12635215
Q7SHS0	ABC transporter	4,56113187	0,00066013	0,0167944
Q6MVM8	Related to subunit of VP52-54 complex	4,55045098	0,0015368	3,00E-02
V5IQ32	Autoinducer 2 sensor kinase/phosphatase luxQ	4,55032271	0,00073543	0,0177196
Q6MVK0	Related to RNA Polymerase I Transcription Factor Rrn3	4,54262533	0,04151805	0,18052218
Q9HE63	RNA polymerase II holoenzyme cyclin-like subunit	4,53055945	0,01956212	0,12425986
Q7S1N7	T-complex 11	4,53052424	0,00180408	0,0325529
Q6MFE4	Conserved oligomeric Golgi complex subunit 3	4,52070868	0,00130746	0,02677671
Q7RXG5	ADA HAT complex component 1	4,51310798	0,02397337	0,13612457
V5IN36	Fungal specific transcription factor	4,50678713	0,02268531	0,13376982
Q873A7	Uncharacterized protein B24N11.170	4,47545643	0,03678484	0,17063499

Q6MFG6	Bms1-type G domain-containing protein	4,47272055	0,03908499	0,17573231
Q7SE39	Phosphatidylinositol:UDP-GlcNAc transferase PIG-C	4,4515706	0,01107035	0,09469391
Q7SBT8	Pentatricopeptide repeat protein	4,44414203	0,00066457	0,01680304
Q1K6H9	RING finger protein	4,39642202	0,0413475	0,18052218
Q1K8K2	Cell cycle control protein	4,39385446	0,02323347	0,13422324
Q7SDF3	ATP-dependent DNA helicase mph1	4,38834406	0,02751009	0,14446325
Q9P4X7	Related to gastric mucin	4,3876384	0,00070429	0,01737806
Q7SHH2	mRNA stability protein	4,35590766	0,00515149	6,15E-02
Q7S0J7	ATP-binding cassette transporter	4,31574304	0,02612826	0,14093637
Q7RYW9	Enoyl-CoA hydratase	4,30801698	0,00121678	0,02545437
Q7RWE3	DNA polymerase epsilon catalytic subunit	4,30184845	0,02362491	0,13496182
Q7SB29	NACHT domain-containing protein	4,29204947	3,96E-05	0,00205139
Q6MUU8	Uncharacterized protein 5C2.100	4,28965708	0,04693982	0,19323165
Q7S3C7	PHM7_ext domain-containing protein	4,27020723	0,0086288	0,08310399
Q1K8N7	Leptomycin B resistance protein pmd1	4,26781929	0,02201767	0,13146409
Q07842	NADH-ubiquinone oxidoreductase 10.5 kDa subunit	4,26666398	0,00071741	1,75E-02
Q7S0H7	Endosome-associated ubiquitin isopeptidase	4,25700584	0,04572506	0,19091728
Q7RUY5	TPR_REGION domain-containing protein	4,24621853	0,03611263	0,16885538
V5IPP0	Hydroxyproline-rich glycoprotein dz-hrgp	4,24381411	0,00499507	0,06050378
Q7SI40	EST1_DNA_bind domain-containing protein	4,22989685	0,00240834	3,91E-02
V5IQQ8	ELMO/CED-12 family protein	4,22844812	0,00174231	0,0319201
Q7SCY7	Protein sym-1	4,22495966	0,04218803	0,18189704
Q7S1J7	ANK_REP_REGION domain-containing protein	4,22495966	0,04218803	0,18189704
Q7SCZ2	Transcription initiation factor TFIID subunit 7	4,22495966	0,04218803	0,18189704
Q873C0	Related to ubiquitin-protein ligase HUL4	4,21810001	0,0070378	0,07487491
Q7SGQ9	Acetamidase	4,21280077	0,00011685	0,00469247
Q7RZY5	MFS transporter	4,20023039	0,00666872	0,07208237
Q9P5P9	Related to a-agglutinin core protein AGA1	4,18846965	0,04685033	0,1930573
Q7S0X4	G-patch domain-containing protein	4,17528606	0,00067559	0,01697675
Q6MUZ0	Uncharacterized protein B12J7.160	4,17463904	0,04299902	0,18365378
Q7S914	DUF221 domain-containing protein	4,16070534	0,0071692	0,07514243
Q7RWZ3	SPX domain-containing protein	4,1556127	0,0104795	0,09152249
Q7RYU7	ZIP metal ion transporter	4,14207295	0,02698708	0,14299364
Q7SDH5	Mannan polymerase complexes MNN9 subunit	4,14189071	0,00014194	5,33E-03
Q7S8L0	Vps52/Sac2 family protein	4,12445835	0,00079818	0,01857594
Q7S6Y5	MBOAT_2 domain-containing protein	4,12140694	0,01383727	0,10437838
Q7SAG6	Galactose oxidase	4,11845675	0,04966711	0,1991495
Q7S5S4	Endosomal peripheral membrane protein	4,10764487	0,0009139	0,02068138
U9W3C8	Sucrose transporter	4,10546789	0,03035587	0,15111104
Q1K6L9	CDT1_C domain-containing protein	4,10323203	0,02878556	0,14689848
Q96UD5	Uncharacterized protein 17E5.030	4,08944838	0,01496053	0,10962137
A0A0B0E761	RING-type domain-containing protein	4,08944838	0,01496053	0,10962137
F5HDM5	RING-type domain-containing protein	4,08944838	0,01496053	0,10962137
Q92247	Putative oxidoreductase bli-4, mitochondrial	4,07684796	0,0464621	0,19236501
Q7SBI9	Choriogenin Hminor	4,07432149	0,00020276	0,00709818
Q1K7X4	MARVEL domain-containing protein	4,06631655	0,00370679	0,04978033
Q7RXX4	F-box domain-containing protein	4,06551225	0,01842124	0,1205326
Q7S2I4	Serine threonine protein kinase	4,0538708	0,00229139	0,03841301
Q7S5G6	GTPase activating protein Sar1	4,05223768	7,15E-05	0,00314717
Q7S8V9	Autophagy-related protein 33	4,04708192	0,00919251	0,08554972

V5IKF7	AAA_16 domain-containing protein	4,03447216	0,04890526	0,19755022
Q7RY49	GTPase-activating protein gyp3	4,03198849	0,00174963	0,0319201
V5IM27	Exocyst complex component Sec3	4,02987124	2,16E-05	0,00124574
Q7SFZ1	Spc7 domain-containing protein	4,00832811	0,00609811	0,06824554
Q1K891	F-box domain-containing protein	4,00351692	0,00444048	5,60E-02
Q9P8G5	Putative SNARE protein	4,00215334	0,00040626	0,01163668
Q7S1C4	Integral membrane protein	4,00090327	0,00307809	0,046019
Q7RZD5	ARID domain-containing protein	3,99890149	0,0014379	0,02861364
Q7SEY6	Bromodomain-containing protein	3,99656113	0,01105504	0,09469391
Q7RZM0	HEAT repeat protein	3,9957987	0,00065062	0,01665586
Q86ZI4	Pyruvate decarboxylase	3,98616803	0,03255449	0,15780261
Q7RYV6	Mis12 domain-containing protein	3,955173	0,03785289	0,17293009
Q1K7X8	Zn(2)-C6 fungal-type domain-containing protein	3,95043525	0,00664364	0,07208237
Q7SDI9	LIM domain-containing protein	3,94902311	0,00455764	0,05708899
Q9P530	Related to YRO2 protein	3,92433068	0,01878152	0,121531
Q7SH04	Origin recognition complex subunit 1	3,92152575	0,01342496	0,10306245
Q7S4N3	SMC_N domain-containing protein	3,91645077	0,01031922	0,09109381
Q7S8I5	CTLH domain-containing protein	3,89864139	0,03872058	0,17447688
Q872S6	Related to sugar transport protein STP1	3,86934281	0,01511107	0,11013336
Q7SA35	Dol-P-Glc:Glc(2)Man(9)GlcNAc(2)-PP-Dol alpha-1,2-glucosyltransferase	3,85331521	0,03968001	0,1764705
Q7S878	GH16 domain-containing protein	3,84904495	0,0357232	0,16741676
Q873H2	Related to stomatin	3,84246518	0,00843289	0,08241739
V5IP84	Glycerol:H+ symporter	3,83612972	0,02306319	0,13389264
Q7RYT7	DUF2428 domain-containing protein	3,83072402	0,0245039	0,13690512
Q7SAD6	P-loop containing nucleoside triphosphate hydrolase protein	3,80388509	0,00945017	8,68E-02
Q7SHL0	Flavin-binding monooxygenase	3,79432788	0,00339987	0,04769129
Q1K862	DNA replication licensing factor MCM5	3,76760323	0,01052081	0,09165746
Q1K618	Mitochondrial ATPase	3,7661617	0,00414031	0,05383719
Q7SEI5	Cell morphogenesis protein	3,7632481	0,02301844	0,13389264
Q6M9I5	Related to GTP-binding protein FZO1, required for biogenesis of mitochondria	3,7440897	0,00435291	0,05537123
Q7S1T6	Structural maintenance of chromosomes protein 4	3,73752368	0,01346153	0,10306245
P47950	Mitochondrial protein cyt-4	3,71546829	0,01318622	0,10287762
Q9P6Z0	Uncharacterized protein 13E11.260	3,71391112	0,01124036	0,09512505
Q7RW96	Heat shock protein 30	3,7064448	0,01528494	1,11E-01
Q7S358	ArAE_2_N domain-containing protein	3,70448729	0,04754566	0,19474703
Q7SEA5	Ferric reductase	3,70082435	0,00242158	3,92E-02
Q1K8U9	MYND-type zinc finger protein samB	3,6665498	0,00199405	0,03475588
Q6MUV0	Related to cobalt accumulation protein COT1	3,6622499	0,01037909	0,09122913
Q7SFK1	ADP-ribosylation factor family protein	3,65143023	0,00021532	0,00734126
Q1K8L3	Calcium permease	3,64957737	0,00165669	0,03141566
Q7S1F5	CCCH zinc finger and SMR domain-containing protein	3,6436101	0,00213762	0,03678869
Q6MWN4	Uncharacterized protein B22K18.010	3,64191345	0,04116056	1,81E-01
Q7SCX9	PAP2 domain-containing protein	3,62813774	0,00077983	1,85E-02
Q1K7K1	Multidrug resistance-associated protein 5	3,60882934	0,00681649	0,07289906
E7BAR7	Bifunctional lycopene cyclase/phytoene synthase	3,59442101	0,0031134	0,04605633
Q96TZ7	Uncharacterized protein B8L3.060	3,5940857	0,00105045	2,31E-02
Q7SEA4	Copper transport protein	3,59147716	0,0059526	6,74E-02
A7UW66	Dual-specificity kinase	3,58294021	0,00652715	0,07167621

Q9C169	Catalase-3	3,55792309	9,63E-06	0,00074424
Q1K505	Leucine-rich repeat-containing protein sog2	3,55765202	0,02269776	0,13376982
Q68KF7	F-box/WD-40 repeat-containing protein	3,54055031	0,03280076	0,15843388
Q872N8	Uncharacterized protein B19A17.140	3,53871998	0,00855705	0,08266429
Q7SCI8	Alpha-1,6-mannosyltransferase subunit	3,53766561	0,00230703	0,03841301
Q7S1Y8	von Willebrand RING finger domain-containing protein	3,53530056	0,00022141	0,00737305
Q9P3C6	DENN-domain-containing protein	3,53192518	0,00113926	2,46E-02
V5IMM0	Anucleate primary sterigmata protein B	3,53172056	0,0214218	0,12922486
Q6MVH5	Sec20-domain-containing protein	3,52012678	0,03189382	0,15552032
Q7SBU6	Chromatin modification-related protein eaf-1	3,5147951	0,00563929	0,0656208
Q7S4W6	Transmembrane protein UsgS	3,50586251	0,02020957	0,1259945
Q7SF30	VPS9 domain-containing protein	3,4958324	0,00905819	0,08482505
Q7SCL1	GPI-anchored wall transfer protein 1	3,49583189	0,02595234	0,14042378
Q7SI42	Initiation-specific alpha-1,6-mannosyltransferase	3,49429053	0,00854871	0,08266429
Q7S9T7	Oxidoreductase	3,48524037	0,01031192	0,09109381
Q6MGD9	Uncharacterized protein B13C5.160	3,48415913	0,02761143	0,14462458
Q7SEW7	Two-component system response regulator	3,46628923	0,00238832	0,03897431
Q7SF11	Phospholipid-transporting ATPase	3,44152549	0,00015686	0,00584104
Q1K5C2	Pps1 dual specificty phosphatase	3,43475241	0,01173591	0,09759103
A0A0B0DVB5	Vacuolar import and degradation protein 21	3,42984911	0,00589313	0,06705073
Q7S240	Oligopeptide transporter	3,42959113	0,03584745	0,16780703
Q12644	NADH-ubiquinone oxidoreductase 23 kDa subunit, mitochondrial	3,41919444	0,00474636	0,05873438
Q7S0S1	Metal resistance protein YCF1	3,40044291	0,0081006	0,0811248
A0A0B0DPD3	Metal resistance protein YCF1	3,40044291	0,0081006	0,0811248
Q7S8J1	Lysophospholipase nte1	3,39087396	0,00793445	0,0811248
Q7SGN1	Aquaporin	3,388335	0,02561884	0,13980585
Q1MW88	Response regulator-like protein	3,38201203	0,00957431	0,08711394
Q7S8M3	Similar to	3,38154869	0,00424312	0,05465346
Q1K519	Intermembrane space AAA protease IAP-1	3,36071063	0,0478527	0,19495303
Q7RZL8	Peroxin-26 Pex26-Penicillium chrysogenum	3,35393922	0,00902576	0,08479242
A0A0B0E9C0	Peroxin 26	3,35393922	0,00902576	0,08479242
Q7S0J4	NAD(P)-binding protein	3,35278301	0,00495315	0,06020203
Q7S9N1	Purine utilization positive regulator	3,34495262	0,04248286	0,18224992
Q7RW65	Phosphatidylinositol 4-kinase	3,34483211	0,00349587	0,04837529
Q7RYV8	Pentatricopeptide repeat domain-containing protein	3,34483199	0,00681253	0,07289906
Q6MGF1	Related to ser/thr protein kinase KIN4	3,33678711	0,00849688	8,25E-02
Q7S5R2	Mitotic spindle checkpoint component MAD2	3,32570595	0,0108046	0,09316975
Q01397	Dynactin, 150 kDa isoform	3,3200945	0,00034837	0,01054674
Q7S0N4	BTB domain-containing protein	3,30931843	0,00748367	0,07760285
Q7S6A6	Non-specific serine/threonine protein kinase	3,29883077	0,0003826	1,11E-02
V5INZ8	Response regulator, variant	3,29799514	0,00757664	0,07807764
Q7RV27	Zn(2)-C6 fungal-type domain-containing protein	3,291956	0,0057768	0,06665289
U9W303	Filamentation protein	3,26163846	0,00117127	0,02498715
A0A0B0DEA6	RIC1-domain-containing protein	3,2510178	0,00738767	0,07680179
Q1K6R0	AP-2 complex subunit mu-1	3,24222292	0,0092108	0,08554972
A0A0B0DYZ0	Alpha/beta-hydrolase	3,23117825	0,0493571	0,19892911
V5IKQ2	Rhomboid domain-containing protein	3,21856012	0,01765991	0,11800159
Q7S9F7	PAS domain-containing protein	3,20998365	0,00713617	0,07514243
Q9HFW7	Guanine nucleotide-binding protein alpha-3 subunit	3,20541403	0,00343635	0,04803852

Q9P532	Uncharacterized protein B24H17.130	3,1851883	0,00073215	0,0177196
V5INY8	DUF625 domain-containing protein	3,16406232	0,01057416	0,09176218
Q1K861	GARP complex component	3,16248241	0,0031743	0,04660188
Q1K7F7	Phospholipid-transporting ATPase	3,15976938	0,00222852	0,03787559
Q7SDF7	Proteasome endopeptidase complex	3,15528122	0,02638773	0,14158056
U9W385	Ubiquitinyl hydrolase 1	3,15093408	0,00754452	0,07803623
Q7RUV0	C2 domain-containing protein	3,14730785	6,40E-05	0,0029432
Q7S1Z5	Sporulation protein RMD1	3,13192051	0,03308789	0,15907041
Q7S0V6	PEP5	3,1286994	0,00396593	0,05240142
Q7S6D3	Nonribosomal peptide synthase 2	3,12599413	0,01962784	0,12436488
Q7SER5	WD repeat containing protein 23	3,10376719	0,00901987	0,08479242
Q96TZ3	Uncharacterized protein B9B15.005	3,1006528	0,02749355	0,14446325
Q8X082	Replication factor C subunit 5	3,09998861	0,02217044	0,1321836
Q7SBR1	ATP-dependent RNA helicase mrh4, mitochondrial	3,09359577	0,01835566	1,20E-01
Q6MUP4	Uncharacterized protein B22I21.220	3,09342107	0,01951857	0,12425986
Q7S9J9	FNIP_N domain-containing protein	3,08336849	0,01076329	0,09315761
Q7S8M0	RING-type domain-containing protein	3,07862846	0,0030529	0,046019
Q6MGF5	Related to RVS167 protein (Reduced viability upon starvation)	3,06365338	0,01552398	0,11135948
Q872P1	Uncharacterized protein B19A17.110	3,06236689	0,00894417	0,08460814
Q7RU49	ARM repeat-containing protein	3,05606825	0,00572091	0,0661945
Q45KI1	Phytochrome-1	3,04436375	0,01477563	0,10885068
Q7S055	Autophagy-related protein 11	3,03976675	0,00802179	0,0811248
Q1K4S3	Hexose transporter HXT13	3,03896506	0,01910138	0,12285765
Q7SEE6	Trichothecene C-15 hydroxylase	3,03329231	0,01322497	0,10290081
V5IKL0	Vac14_Fig4_bd domain-containing protein	3,02731004	0,03807768	0,17310342
Q7S304	E3 ubiquitin-protein ligase bre1	3,02478673	0,00405764	0,05293023
Q7RYY6	Alpha-actinin	3,01584023	0,01300271	0,10202891
V5ILM4	F-box domain-containing protein	3,00650352	0,00379952	0,05052868
Q7RWB9	Two-component sensor protein histidine protein kinase	3,00641334	0,00012544	0,00498856
Q7S875	NADH-cytochrome b5 reductase	2,99496709	0,02722116	0,14377418
Q7SB06	PH domain-containing protein	2,97503321	0,03662199	0,17026525
Q7S6C9	Similar to	2,96282743	0,0035713	0,04908738
Q1K8Z4	Nucleoporin protein Ndc1-Nup	2,94102232	0,00151528	0,02983935
V5IMU4	VPS9 domain-containing protein	2,9353626	0,02488839	0,13781798
Q7SEH0	Ammonium transporter	2,92929672	0,00485698	0,05975517
Q1K5A5	DUF3492 domain-containing protein	2,92307027	0,00978991	0,08813069
A0A0B0E4V3	Glycosyltransferase family 4 protein	2,92307027	0,00978991	0,08813069
V5IQZ3	Ras GTPase activating protein	2,92252515	0,00083327	0,01928283
Q7RXP0	1-acyl-sn-glycerol-3-phosphate acyltransferase	2,91346325	0,01873502	0,121531
P78714	White collar 2 protein	2,89937563	0,00092053	0,02071708
Q9C2D8	Uncharacterized protein 9G6.270	2,89201567	0,0136403	0,10381975
P19970	Frequency clock protein	2,88426686	0,02995518	0,15017919
Q1K6I5	Calcium-transporting ATPase	2,86259335	0,00323815	0,0468674
Q9C256	RBP11-like subunits of RNA polymerase	2,86166019	0,04530961	0,18995717
Q7S6V7	Serine/threonine-protein kinase TOR	2,8560536	0,01742235	0,1171037
Q7SHX8	Protein efr-3	2,82335617	0,01640698	0,11410825
Q7S7I3	Polarized growth protein Boi2	2,81702146	0,0438205	0,18605557
Q7RYV1	Midasin	2,81499825	0,00225854	0,03806995
Q1K6Y6	Nucleoporin POM34	2,80917962	0,01264638	0,10092799

V5INI8	Trafficking protein particle complex subunit 12	2,80587965	0,00246746	3,92E-02
Q6MV57	Related to zinc finger protein SFP1	2,80429309	0,03099218	0,15257688
Q7S8E9	Galactose oxidase/kelch, beta-propeller	2,79585074	0,02647409	0,14158524
Q7S1V7	Developmental regulator VosA	2,79490902	0,03819571	0,17344749
V5IMW7	SSCRP protein	2,79326974	0,04237198	0,18224992
Q7SBU4	THO complex subunit Tho1	2,79030554	0,0213354	0,12908387
Q1K5R5	ATP-binding cassette protein	2,78137481	4,61E-05	0,00232944
V5IM45	Transcription factor tau subunit sfc1	2,77502225	0,04253685	0,18224992
A0A0B0ED43	Transcription factor tau subunit sfc1	2,77502225	0,04253685	0,18224992
Q7SDJ4	F-box domain-containing protein	2,76115797	0,02818664	0,14556837
Q7SH92	Sorting nexin-3	2,76049467	0,02091837	1,28E-01
Q7S9J4	ATP-dependent RNA helicase dbp-10	2,75624906	0,03845065	0,1738343
Q6M921	Related to DNA repair protein MMS21	2,75111462	0,04389997	0,18605557
Q7SBC5	GDP-mannose transporter	2,74477842	0,04151773	0,18052218
Q7S2E8	Oxidoreductase	2,74160132	0,04387117	0,18605557
Q9C168	Catalase-1	2,73463796	4,38E-05	0,00224048
Q872I5	Chromatin-remodeling ATPase INO80	2,71879119	0,03842616	0,1738343
Q7S632	PrnX protein	2,71606064	0,03094016	0,15250406
O13396	DNA mismatch repair protein msh-2	2,70831008	0,01268992	0,10092799
Q7S4P7	Nuclear import and export protein Msn5	2,70337125	0,0004242	0,01198292
Q1K527	Exocyst complex component Sec6	2,69403818	0,00276976	0,04297324
Q7SER3	UBX domain-containing protein	2,68943433	0,03802935	0,1730758
Q1K6P9	Secretion pathway protein Sls2/Rcy1	2,66551519	0,00595046	0,06735319
Q6MV26	Diacylglycerol O-acyltransferase	2,66046581	0,03401995	0,16146667
Q7S0A0	Chorismate synthase	2,65364967	0,03949386	0,17631918
Q7SF97	Myosin type-2 heavy chain 2	2,65008497	0,00884997	0,0843011
Q7SB56	Rab-GAP TBC domain-containing protein	2,64298593	0,02331176	0,13448586
Q7S5H0	Nucleolar protein 9	2,6394616	0,00932559	0,08594301
Q7RX31	Adaptin ear-binding coat-associated protein 2	2,62431619	0,01432912	1,07E-01
Q1K8Q9	Mitochondrial AAA ATPase	2,62198575	0,00607439	0,06822124
Q7SEG8	TPR_REGION domain-containing protein	2,60645578	0,02273201	0,1337792
Q7S405	Similar to	2,6063512	0,00279029	0,04298356
Q7S1I4	Condensin complex subunit 1	2,60393135	0,00347655	0,04827096
V5IRD3	Nucleoporin-9	2,59105651	0,00269243	0,04209241
P45443	Dynein heavy chain, cytoplasmic	2,58767904	5,83E-05	0,00280998
Q7SBA5	DUF803 domain membrane protein	2,57963459	0,03354444	0,16051172
Q1K7G1	Hatpase c domain-containing protein	2,57369083	0,01821122	0,11979373
Q1K8Z5	Intracellular protein transporter	2,57159747	0,04745244	0,19455977
A0A0B0DT44	Conserved oligomeric Golgi complex subunit 4	2,55484495	0,02088236	0,12790417
Q7RVS8	Calcium/calmodulin-dependent protein kinase type I	2,55236653	0,02755287	0,14450264
Q01631	Adenylate cyclase	2,55127066	0,01422902	0,10650313
Q7SH99	Syntaxin 5	2,54838417	0,01164672	0,09715881
Q9HE88	Probable exocyst complex component sec8	2,54755955	0,00356946	0,04908738
Q1K8S9	Sphingosine-1-phosphate lyase	2,54435669	8,88E-06	0,00071666
Q6MVW2	Uncharacterized protein B15B3.020	2,53193179	0,04180615	0,18107847
Q7SA31	FAD/NAD(P)-binding domain-containing protein	2,52820882	0,02817587	0,14556837
Q7SBB6	Probable Delta(7)-sterol 5(6)-desaturase	2,52593511	0,03301606	0,15907041
Q7S0F6	Transcription factor	2,52088522	0,04334195	0,18463597
Q7SG21	Endoplasmic reticulum transmembrane protein	2,51733574	0,02027221	0,1261686
Q7SBA7	Nuclear condensin complex subunit 3	2,50654912	0,0261503	0,14093637

Q7S9L2	Protein stu-1	2,50581464	0,0207366	0,12753321
Q7S8B6	Exocyst complex component exo84	2,50552516	0,00102426	0,02267758
Q6MUZ9	Dolichyl-diphosphooligosaccharide--protein glycotransferase	2,49267112	0,0114346	0,09617275
Q7S4G8	TRAPPC10 domain-containing protein	2,48399353	0,04466495	0,18821772
Q7RYL2	Exocyst complex component SEC15	2,47950024	0,01378643	0,10418677
Q7RXB7	Conserved oligomeric Golgi complex subunit 5	2,47272003	0,03842769	0,1738343
Q7SGE1	Ribosome biogenesis protein nsa2	2,46291474	0,03393163	1,61E-01
Q7RZL3	Phospholipid-transporting ATPase	2,44952444	0,01226246	0,0992629
Q7RWL9	Niemann-Pick C1 protein	2,44162887	0,01119037	0,09489807
V5IM93	DNA topoisomerase 2	2,44037966	0,02967688	0,14982849
Q7SH40	LIM and Rho-GAP domain-containing protein	2,43021554	0,01188914	0,09759103
V5INW6	DUF221 domain-containing protein	2,42390625	0,01682072	0,11591125
Q7S291	MYND-type domain-containing protein	2,41794453	0,049836	0,1991495
Q7S8Q2	Zinc binuclear cluster-type protein	2,41395732	0,0034573	0,04816703
Q7S1T3	Zn(2)-C6 fungal-type domain-containing protein	2,41278839	0,02369124	0,13515226
E7BAR8	Bifunctional lycopene cyclase/phytoene synthase	2,40673255	0,04070002	0,17908263
Q7SCJ6	Transmembrane alpha-helix domain-containing protein	2,40511713	0,0480374	0,19519959
Q7SCS0	Condensin complex subunit 2	2,39527678	0,01148264	0,09637892
Q7S9M2	Structural maintenance of chromosomes protein	2,3780298	0,02042117	0,12635215
Q1K905	Calcium-transporting ATPase	2,36484816	0,01367731	0,10381975
V5IMJ5	Peroxin-24 Pex24-Penicillium chrysogenum	2,36042919	0,02948671	0,1491284
Q7S4P8	Zn(2)-C6 fungal-type domain-containing protein	2,34253501	0,04597391	0,19156574
U9W4W6	GTP binding protein	2,33770247	0,02253146	0,13336541
Q7S406	Flotillin domain-containing protein	2,32655024	0,0071383	0,07514243
U9W323	CCAAT-binding transcription factor subunit AAB-1	2,32100849	0,03760098	1,72E-01
Q8NJW7	G-protein beta subunit	2,31451396	0,01337425	0,10297393
Q7S3A1	Dopey domain-containing protein	2,27983444	0,01863297	0,12131139
Q7S9K7	Exocyst complex component SEC5	2,27504976	0,01012824	0,09031734
Q6MGJ1	Related to ECM29 protein	2,27094586	0,0046676	0,05811095
Q6MWN2	Related to dock180 protein	2,26774075	0,03716877	0,17144513
Q1K7U1	Cell division control protein 15	2,26721182	0,01820546	0,11979373
Q7S808	Zn(2)-C6 fungal-type domain-containing protein	2,25180068	0,00330685	0,04715479
V5IQG0	Rtg2-like protein	2,23277685	0,0133148	0,10290081
A0A0B0DME5	Ppx-GppA domain-containing protein	2,23277685	0,0133148	0,10290081
Q6M9H2	Related to transcription factor snf5p	2,22308688	0,00416384	0,05387529
A0A0B0E9Z8	SRP54-domain-containing protein	2,21999144	0,0367225	0,17053894
Q7SEN2	CorA family metal ion transporter	2,21163645	0,04718245	0,19391543
Q6MFE8	Bromo domain-containing protein	2,210543	0,0339799	0,16146364
Q05424	Guanine nucleotide-binding protein alpha-2 subunit	2,19807646	0,00558435	0,06516668
A0A0B0DQH8	Guanine nucleotide-binding protein alpha-2 subunit	2,19807646	0,00558435	0,06516668
Q7S6N5	HECT-type E3 ubiquitin transferase	2,18701064	0,01000913	0,0895394
Q7S7L0	Transcriptional co-activator	2,18619569	0,0211915	0,12884425
Q1K4U8	Cyclin-dependent protein kinase complex component	2,18241504	0,01689336	0,11591125
Q7RWZ8	Ras-GAP domain-containing protein	2,180876	0,01508675	0,11013336
Q7RXM3	SIR2 family histone deacetylase	2,17068682	0,04416715	0,18688909
Q6MUQ7	Probable rho3 protein	2,16599154	0,01212291	0,09891525
Q7SB48	ANK_REP_REGION domain-containing protein	2,16383867	0,00567145	0,06580814
Q7RZP0	Target of rapamycin complex 2 subunit sin1	2,15962785	0,04421475	0,18689742
Q6MFS1	Exocyst complex protein exo70	2,14365402	0,0163418	0,11410825

Q7SGP6	Rhodopsin family protein	2,13664088	0,04459593	0,18812044
Q7S4V2	Ubiquitin carboxyl-terminal hydrolase 2	2,1317009	0,02891814	0,14732425
Q7SD81	Exocyst complex component Sec10	2,11529857	0,00339	0,04769129
V5IRK0	Chromodomain helicase	2,08520119	0,02009193	0,12564359
P53659	V-type proton ATPase subunit d	2,06002867	0,01683534	0,11591125
Q7SDJ7	C6 transcription factor	2,04277369	0,00894099	0,08460814
A0A0B0DQT0	Meiotically up-regulated gene 70 protein (Fragment)	2,03810541	0,00130647	0,02677671
Q7SCR6	1-phosphatidylinositol 4-kinase	2,02793804	0,01820589	0,11979373
Q1K5K5	Meiotically up-regulated 190 protein	2,01585713	0,02242518	0,13312584
Q1K809	Proteophosphoglycan ppg4	2,01534681	0,04566337	0,19085425
V5INJ9	DEAD/DEAH box helicase	2,01498446	0,04147267	0,18052218
Q7SHS5	Cell division control protein 54	2,0138901	0,00822002	0,08192019
Q96U60	Probable kinetochore protein ndc80	1,9969354	0,02399462	0,13612457
V5IMF6	Developmental regulator flbA, variant	1,9914814	0,02611794	0,14093637
A0A0B0EFB3	PCI-domain-containing protein	1,99133248	0,04661449	0,19266695
Q7S0D4	Ydr124wp-like protein	1,97732321	0,0242425	0,13651156
Q7RYK2	DUF747-domain-containing protein	1,97356722	0,02491533	0,13781798
Q1K5B2	AP-2 complex subunit alpha	1,95798835	0,04182135	0,18107847
Q7S582	Acyl-CoA dehydrogenase	1,95608798	0,03114416	0,15264803
Q9P5S5	Uncharacterized protein B5O22.270	1,95004577	0,04064993	0,17908263
U9W4I4	Thiamine-4	1,93724315	0,01925193	0,12321238
Q7RVZ1	Actin-interacting protein	1,90882174	0,00431868	0,0551069
Q7S2X7	HEAT repeat containing protein	1,87328036	0,03060953	0,1517877
V5IQ80	Bacilysin biosynthesis oxidoreductase bacC	1,86636578	0,01361432	0,10381975
Q7SAE8	INCENP_ARK-bind domain-containing protein	1,85587766	0,0371373	0,17144513
Q7SD07	DNA replication licensing factor MCM7	1,85549432	0,03391562	0,16142153
Q7SDY4	AMI1 protein	1,85491275	0,02875793	0,14689848
Q1K8B3	NARG2_C domain-containing protein	1,84515867	0,04916568	0,19840651
Q7S2W9	Poly(A)-specific ribonuclease	1,84096449	0,04788153	0,19495303
Q6M9I1	Related to Na+/H+ antiporter CNH1	1,82130224	0,04548136	0,19028769
U9W3M5	TBP associated factor	1,81157528	0,03054515	0,15166471
Q7S1S1	Transcription initiation factor TFIID complex 60 kDa subunit	1,78312684	0,0213775	0,12914786
Q1K506	Calcium-transporting ATPase 3	1,76289645	0,00777623	0,07982814
A0A0B0DUH0	ARID domain-containing protein	1,75552822	0,03799971	0,1730758
Q7RVY6	MAPKK kinase	1,7514833	0,04625408	0,19196184
A0A0B0E5J0	Protein kinase byr2	1,7514833	0,04625408	0,19196184
Q05425	Guanine nucleotide-binding protein alpha-1 subunit	1,74966914	0,04392474	0,18605557
Q1K6F5	Bactericidal permeability-increasing protein	1,74372749	0,04091198	0,17960931
Q7S4Y3	Rho guanyl nucleotide exchange factor	1,71962191	0,02783082	0,14484755
Q7S211	Glycerol-3-phosphate O-acyltransferase	1,70825188	0,04886215	0,19755022
E7BAS2	Bifunctional lycopene cyclase/phytoene synthase	1,6878703	0,02734563	0,14396876
Q7SCS6	Mitotic control protein dis3	1,68348934	0,04796184	0,19508609
Q7S859	KH domain-containing protein	1,68001343	0,00587472	0,06702742
Q7SAX4	Protein transporter sec72	1,66359837	0,0161007	0,11370424
Q873C7	RuvB-like helicase 2	1,66193249	0,01776824	0,11833935
Q6MW49	Related to bile acid ABC transport protein	1,65707714	0,03934856	0,17595519
V5IPF6	Rab18/RabC-family small GTPase, variant	1,65590341	0,02657189	0,1415936
Q7RVT5	SCD domain-containing protein	1,64849967	0,02732938	0,14396876
Q7RYF4	DNA-directed RNA polymerase subunit beta	1,63066179	0,00328142	0,0469954

Q1K7M7	Protein CASP	1,5798465	0,02821511	0,14556837
P37295	Bifunctional lycopene cyclase/phytoene synthase	1,57414335	0,02498445	0,13781798
Q1K7U8	Structural maintenance of chromosomes protein	1,55733131	0,02990531	0,15017919
Q7RY29	Spindle pole body component	1,53032709	0,02508845	0,13812138
P22126	Protein ras-1	1,51452506	0,02172658	0,13029583
Q7S0A7	1,3-beta-glucan synthase	1,50810379	0,01175832	0,09759103
Q871M3	Probable myosin MYO2	1,50185055	0,03502851	0,16491586

log FC: log fold change, FDR: fold discovery rate, n = 5 and 8 for FGSC #2489 (*wt*) and FGSC #11866, respectively.
